# Immune Infiltrates in Breast Cancer: Recent Updates and Clinical Implications

**DOI:** 10.3390/cells10020223

**Published:** 2021-01-23

**Authors:** Maria Vittoria Dieci, Federica Miglietta, Valentina Guarneri

**Affiliations:** 1 Department of Surgery, Oncology and Gastroenterology (DISCOG), University of Padova, 35128 Padova, Italy; federica.miglietta@iov.veneto.it (F.M.); valentina.guarneri@unipd.it (V.G.); 2 Medical Oncology 2, Istituto Oncologico Veneto IOV-IRCCS, 35128 Padova, Italy

**Keywords:** breast cancer, immune infiltrate, immune biomarker, tumor-infiltrating lymphocytes, PD-L1, immunotherapy

## Abstract

In recent decades, the increasing interest in the field of immunotherapy has fostered an intense investigation of the breast cancer (BC) immune microenvironment. In this context, tumor-infiltrating lymphocytes (TILs) have emerged as a clinically relevant and highly reproducible biomarker capable of affecting BC prognosis and response to treatment. Indeed, the evaluation of TILs on primary tumors proved to be strongly prognostic in triple-negative (TN) BC patients treated with either adjuvant or neoadjuvant chemotherapy, as well as in early TNBC patients not receiving any systemic treatment, thus gaining level-1b evidence in this setting. In addition, a strong relationship between TILs and pathologic complete response after neoadjuvant chemotherapy has been reported in all BC subtypes and the prognostic role of higher TILs in early HER2-positive breast cancer patients has also been demonstrated. The interest in BC immune infiltrates has been further fueled by the introduction of the first immune checkpoint inhibitors in the treatment armamentarium of advanced TNBC in patients with PD-L1-positive status by FDA-approved assays. However, despite these advances, a biomarker capable of reliably and exhaustively predicting immunotherapy benefit in BC is still lacking, highlighting the imperative need to further deepen this issue. Finally, more comprehensive evaluation of immune infiltrates integrating both the quantity and quality of tumor-infiltrating immune cells and incorporation of TILs in composite scores encompassing other clinically or biologically relevant biomarkers, as well as the adoption of software-based and/or machine learning platforms for a more comprehensive characterization of BC immune infiltrates, are emerging as promising strategies potentially capable of optimizing patient selection and stratification in the research field. In the present review, we summarize available evidence and recent updates on immune infiltrates in BC, focusing on current clinical applications, potential clinical implications and major unresolved issues.

## 1. Introduction

**Figure 1:**
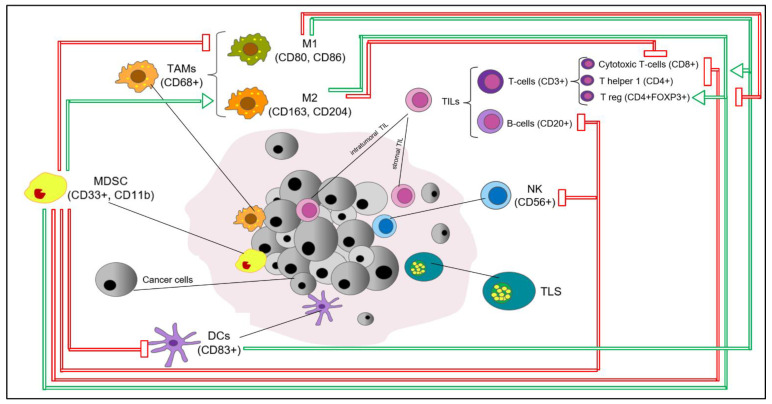
Key immune cell subsets in breast cancer tumor microenvironment. The production of IFNγ by CD4+ Th1 cells mediates the expansion, differentiation and activation of CD8+ tumor-infiltrating lymphocytes (TILs), which subsequently release cytotoxic cytokines and directly kill cancer cells (via recognition of specific tumor-associated antigens on the surface of antigen presentation cells (APCs) or cancer cells); CD4+FOXP3+ TILs represent immunosuppressive mediators through the inhibition of CD8+ T cells, CD4+ Th1 cells, APCs and natural killer cells (NKs); M1 tumor-associated macrophages (M1-TAMs) are associated with Th1 cytotoxic immune response, thus exhibiting antitumor properties; M2-TAMs contribute to the activation of Th2 immune response, thus showing an immunosuppressive role (e.g., suppression of T cell function); NKs are cytotoxic members of the innate immune system (release of cytotoxic cytokines and direct killing of cancer cells); dendritic cells (DCs) are antigen-presenting cells and are crucial players of the adaptive immune system; myeloid-derived suppressor cells (MDSCs) represent immature myeloid cells (possibly originated from bone marrow precursors) with an immunosuppressive function via the inhibition of T cells, B cells, NKs, M1-TAMs and DCs. The recruitment and accumulation of immunosuppressive mediators into the tumor bed is mediated by the secretion of cytokines and chemokines (e.g., IL6, IL1-β, TGF-1β, CCL2) by tumor cells. Red and green arrows reflect inhibitory and stimulatory relationships, respectively. Abbreviations: IFNγ, interferon gamma; TILs, tumor-infiltrating lymphocytes, APCs, antigen presentation cells, NKs, natural killer cells, DCs, dendritic cells, TAMs, tumor-associated macrophages; MDSCs, myeloid-derived suppressor cells; TLS, tertiary lymphoid structure.

Breast cancer (BC) is not considered a highly immunogenic tumor type, especially if compared with melanoma or lung cancer. However, in recent decades, it has been consistently reported that the BC tumor microenvironment (BC-TME) encompasses a wide range of cell populations of both the innate and adaptive immune systems, which have been reported to be biologically/clinically relevant to varying degrees [1], as summarized in Figure 1. Immune infiltrates in BC encompass immune cells both directly in contact with tumor cells, known as “intratumoral” (int), and within the surrounding stroma, known as “stromal” (str). The evaluation of BC immune infiltrates relies on, among other things, morphological evaluation of immune cells on H&E-stained tumor samples, immunohistochemical staining for specific subsets of immune cells evaluated by classic semiquantitative scoring or by digital pathology, multiplexed fluorescent immunohistochemistry with multispectral imaging that simultaneously identifies and quantifies multiple immune cell subsets in a single formalin-fixed paraffin-embedded (FFPE) slide and provides information on the distribution of immune infiltrates, flow cytometry on fresh tissue and computational tools for immune cell quantification from transcriptomics data.

BC immunogenicity is highly heterogeneous, with different BC subtypes showing different degrees of immune infiltration. In detail, the most biologically aggressive subtypes, namely, triple-negative BC (TNBC) and HER2-positive (HER2+) BC, are characterized by high genomic instability and tumor mutational burden (TMB), both fueling the generation of neoantigens, ultimately fostering the antitumor immune activity. However, an inverse association between TMB/genomic heterogeneity and levels of immune infiltrates in TNBC subtypes has recently been suggested, with immune-rich tumors showing lower degrees of clonal genomic heterogeneity, lower neoantigen loads and somatic mutations and fewer somatic copy number alterations [2]. While seemingly counterintuitive, this observation may reflect the elimination of immunogenic clones by an effective antitumor immune surveillance, thus resulting in lower clonal genomic heterogeneity. Conversely, higher clonal heterogeneity may reflect the escape phase of the immunoediting process, where the selection of cancer clones results in reduced immunogenicity.

Both cytotoxic treatments and anti-HER2 agents are known to be capable of further activating the immune system through immunogenic cell death and antibody-dependent cellular cytotoxicity (ADCC), respectively. In addition, in HER2+ BC, oncogene addiction may trigger the immune system, with HER2 itself acting as a tumor-associated neoantigen [3]. Hormone receptor-positive (HR+)/HER2-negative (HER2−) BC, also known as luminal-like BC, is traditionally considered to be less immunogenic than TNBC and HER2+ BC, given the lower genomic instability and mutational load [3,4,5]. However, available evidence suggests that the immunogenicity of this BC subtype may rely on subtler mechanisms reflecting the complex and dynamic relationship between HR+ BC cells, inflammatory mediators, estrogen levels, endocrine treatments and menopausal status [6].

In the present review, we summarize available evidence on immune infiltrates in BC, focusing on the most recent updates as well as current and potential clinical implications.

## 2. Adaptive Immunity

### 2.1. Tumor-Infiltrating Lymphocytes (TILs)

In recent decades, the morphological evaluation of immune infiltrates in BC has gained tremendous interest in the light of accumulating high-quality evidence supporting TIL clinical validity in BC.

The prevalence of TILs is heterogeneous across different BC subtypes, with TNBC and HER2+ BC typically exhibiting greater TIL infiltration as compared to the luminal-like BC subtype. In detail, a systematic review of 15 studies, including almost 14,000 BC patients, showed that TNBC is the most frequently infiltrated by TILs, with a 20% prevalence of lymphocyte-predominant BC (LPBC), followed by HER2+ BC (LPBC: 16%), with the luminal-like BC subgroup (HR+) showing the lowest degree of TIL infiltration, as well as the lowest prevalence of LPBC (6%) [7]. These observations have been further confirmed in the context of a pooled analysis of six randomized clinical trials including 3771 BC patients receiving neoadjuvant combination chemotherapy, where the authors reported significantly higher proportions of primary tumors with high TILs (≥60%) among TNBC (30%) and HER2+ (~20%) BC patients, as compared to the luminal-like carcinoma subgroup (13%) [8].

An additional source of heterogeneity is represented by the disease setting. Indeed, it has been suggested that TIL infiltration tends to weaken throughout the natural history of BC from the early to advanced stages. In particular, it has been consistently reported that overall TIL levels are not only lower in patients with advanced disease as compared to the early setting, but also in heavily treated advanced BC patients as compared to those treated in the first-line setting for their metastatic disease [9,10,11,12]. Moreover, heterogeneity in TIL levels has also been observed within different sites of BC metastases, with the lungs showing the highest degree of TIL infiltration, while the liver and skin show the lowest [11,13,14] (Figure 1).

The ever-growing interest towards the evaluation of TILs fostered the development of the International Working Group on Immuno-Oncology Biomarkers, aiming at providing a standardized methodology for TIL assessment in BC samples, in order to improve consistency and reproducibility across studies, in preparation for TIL clinical implementation, also given the endorsement of TIL quantification and reporting in TNBC and HER2+ BC by the St Gallen Consensus Conference (TNBC), WHO (both TNBC and HERBC2+) and ESMO 2019 Guidelines [15,16]. In this context, driven by the increasing need for a reliable and accurate quantitative assessment of TILs by pathologists, the International Working Group on Immuno-Oncology Biomarkers conducted the so-called RING studies [17]. In detail, in the RING studies, the inter-pathologist reproducibility of TIL measurement on untreated BC samples was formally evaluated, providing strong evidence that TILs may be accurately and reliably scored, especially with the support of software-guided slide viewers. Similarly, encouraging results have been obtained in a subsequent RING study testing the interobserver agreement in TIL assessment on residual disease (RD) after neoadjuvant chemotherapy [18].

Through these joint efforts, recommendations for TIL assessment on primary BC, RD after neoadjuvant chemotherapy, ductal carcinoma in situ and metastatic sites from BC patients are currently available (www.tilsinbreastcancer.org).

#### 2.1.1. TIL Clinical Relevance in the Early Setting According to BC Phenotype

##### TNBC

The most robust body of evidence on the clinical relevance of TILs concerns early TNBC.

The role of pre-therapeutic TILs has been extensively investigated in the adjuvant setting. In particular, a pooled analysis of individual patient data from nine large clinical studies (n = 2148) was performed, seeking to provide conclusive data on the prognostic value of TILs in early TNBC patients receiving standard anthracycline-based adjuvant chemotherapy [19]. The quantity of strTILs, assessed by standard methodology [20], was shown to be positively and significantly associated with survival in terms of invasive disease-free survival (iDFS), distant DFS (DDFS) and overall survival (OS), with a 14%, 17% and 17% reduction in the risk of events, respectively, for each 10% increase in TILs. Moreover, strTILs proved to add independent prognostic information beyond those provided by traditional clinicopathologic features, including age, tumor size and nodal burden, thus suggesting that the inclusion of TILs in an integrated clinicopathologic prognostic model may further refine our ability to prognostically stratify early TNBC patients undergoing standard adjuvant chemotherapy. When adopting 30% as the cutoff to differentiate patients with high vs. low TILs, excellent survival rates were observed in patients with high TIL infiltration, especially in the node-negative (N0) subpopulation. These results raised the appealing possibility to identify early TNBC patients with excellent prognosis based on high TIL levels, who might deserve de-escalated adjuvant systemic treatments. In this context, promising data come from a retrospective study including 605 early TNBC patients, of which 182 did not receive any adjuvant cytotoxic treatment [21]. The authors reported that higher TIL levels were significantly associated with improved iDFS and OS rates in systemically untreated early TNBC patients. Interestingly, the risk for an iDFS event appeared to be halved in patients showing an LPBC phenotype as compared to those with lower TILs. A pooled analysis of individual patient data from one prospective randomized clinical trial and three single-institution retrospective series (n = 476) was conducted in order to evaluate the intrinsic prognostic role of TILs [22]. strTILs were found to be positively, significantly and independently associated with survival in terms of iDFS, DDFS and OS. In particular, in the multivariate analysis, each 10% TIL increment corresponded to a 10%, 14% and 12% risk reduction for iDFS, DDFS and OS events, respectively. Interestingly, by adopting the previously suggested cutoff of 30% of TILs, outstanding survival rates were observed in early TNBC patients with stage I (according to 8th edition AJCC) disease exhibiting high TIL levels, with estimated 5-yy iDFS, DDFS and OS of 97%, 95% and 98%, respectively. The prognostic value of TILs in the absence of adjuvant chemotherapy has been recently demonstrated in a retrospective Dutch cohort of 481 young patients (<40 years) with T1-3/N0 TNBC, treated with locoregional therapy only in the early setting [23]. The 15-yy OS rates in patients with low vs. intermediate vs. high TILs (by adopting 30% and 75% as cutoffs) were 59% vs. 76% vs. 93%, respectively, with a very low incidence of DDFS events in the high TIL subgroup (15-yy DDFS in low vs. intermediate vs. high TILs: 39%, 95% CI 36–42% vs. 16%, 95% CI 12–19% vs. 1.9%, 95% CI 0–3.4%). Overall, these data suggest TILs as a promising tool capable of identifying a subgroup of early TNBC patients characterized by excellent absolute prognosis, for whom adjuvant systemic therapy might be safely withheld. In this context, clinical trials testing de-escalated adjuvant treatment strategies based on strTIL levels are urgently needed.

The clinical relevance of TILs has been also investigated in TNBC patients receiving neoadjuvant chemotherapy, where an association between TILs and both response to treatment and prognosis has been reported. In particular, in a pooled analysis of individual patient data from six randomized clinical trials of combination neoadjuvant chemotherapy (n = 3771) [8], strTILs were quantified in the pre-therapeutic BC samples according to international guidelines [20]. In the TN cohort (n = 906, 25%), higher TILs were shown to be significantly associated with increased pathologic complete response (pCR) rates when considered both as a continuous and categorical variable. In particular, pCR rates in low vs. intermediate vs. high TILs were 31% vs. 31% vs. 50%, respectively. In addition, a significant and independent association between increased TIL levels and both DFS and OS was reported.

Overall, the available evidence has allowed TILs to reach level 1b evidence for their clinical validity as a prognostic marker in early TNBC. Indeed, the 16th St Gallen International Breast Cancer Consensus Conference [15], WHO (http://publications.iarc.fr) and ESMO (2019 Clinical Practice Guidelines [16]) endorsed routine quantification and reporting of TILs in early TNBC samples. However, it should be noted that the use of TILs as a tool to guide systemic treatment choices in the early setting in TNBC patients is currently not recommended [15].

A further refinement of TIL clinical relevance comes from studies evaluating the prognostic role of TILs on RD after neoadjuvant chemotherapy. Indeed, the achievement of pCR after neoadjuvant chemotherapy represents a surrogate endpoint for long-term outcome. However, its prognostic value should be considered suboptimal, since a proportion of patients achieving pCR will eventually relapse and the presence of RD after neoadjuvant chemotherapy does not necessarily translate into poor prognosis. In this context, accumulating evidence supports TILs evaluated in the RD (RD-TILs) as a promising tool potentially capable of further stratifying TNBC patients with RD after neoadjuvant chemotherapy. In detail, a retrospective analysis of 278 TNBC patients with RD after neoadjuvant chemotherapy showed a significant correlation between RD-TILs and both metastasis-free survival (MFS) and OS [24]; in particular, for each 10% strTIL increment, a reduction of 21% in the risk of metastasis or death was observed. Similar results were observed when considering intratumoral TILs (intTILs), with a 22% reduction in the risk of metastasis or death for each 10% intTILs increment. A significant association between RD-TILs and prognosis was also confirmed when considering the dichotomization between high and low TILs, by adopting 60% as the cutoff. In particular, 5-yy MFS and OS rates for high TILs vs. low TILs were 81.5% and 91% vs. 46% and 55%, respectively. Interestingly, the magnitude of the RD-TIL prognostic value was greater in patients with node-positive and/or RD ≥ 2 cm. In addition, by comparing pre-therapeutic and post-neoadjuvant TIL levels in patients failing to achieve pCR, in all but one cases, baseline TILs were lower than post-neoadjuvant surgical samples, thus confirming the preclinical observations that chemotherapy may induce lymphocytes attraction [25,26]. These findings were further confirmed in a subsequent retrospective study including four TNBC patients cohorts (n = 475) failing to achieve pCR after neoadjuvant chemotherapy [27]. Indeed, higher RD-TILs were significantly and positively associated with both recurrence-free survival (RFS) and OS, with a 14% and 13% reduction in the risk of events, respectively. In addition, multivariate analysis revealed that RD-TILs provided additional and independent prognostic information beyond that provided by residual cancer burden (RCB) alone, which represents a composite score evaluated on RD, encompassing the pathologic measurements of the primary tumor in terms of size and cellularity, as well as nodal burden in terms of number of positive nodes and largest metastasis dimension. The prognostic value of RD-TILs was particularly evident in patients showing intermediate RD burden (RCB class II). These findings overall suggest that a more comprehensive evaluation of RD after neoadjuvant chemotherapy, encompassing not only invasive disease burden but also immunological aspects, may help in better refining patient prognostic stratification among TNBC patients with RD after neoadjuvant chemotherapy, with appealing possible implications in drug development and proper placing. In this context, the presence of RD currently represents a well-established stratification factor for the identification of high-risk patients who may benefit from escalated treatment strategies in the post-neoadjuvant setting. Indeed, the strategical approach of targeting a selected group of patients at high risk of relapse based on the presence of RD after neoadjuvant chemotherapy has recently been endorsed by the FDA [28] for a more rational clinical positioning of investigational anticancer agents. For this reason, the identification of additional stratification factors to be integrated with simple dichotomization in pCR vs. non-pCR may be crucial in this process, with the aim of increasing our abilities to identify patients who may deserve novel or escalated post-neoadjuvant strategies.

##### HER2+ BC

In recent years, the evaluation of immune infiltrates in HER2+ BC has progressively gained momentum [29]. In this context, the prognostic value of TILs in early BC patients receiving adjuvant treatment has been extensively investigated. In detail, the translational analysis of the Finher trial, which randomized 232 early HER2+ BC patients to receive either trastuzumab or no trastuzumab in combination with adjuvant chemotherapy, revealed a positive and significant association between increasing TILs and more favorable DDFS only in trastuzumab-treated patients, thus suggesting a possible predictive role of TILs in this regard [30]. Instead, an opposite association between TILs and benefit from adjuvant anti-HER2 therapies was observed in the N9831 trial, where strTILs were significantly related to better RFS only in patients treated with chemotherapy alone, while no impact was observed in patients receiving adjuvant trastuzumab [31]. However, the whole transcriptome analysis from the same trial revealed that among patients receiving trastuzumab, only those with immune gene-enriched tumors experienced increased RFS following adjuvant treatment, while no predictive effect was observed in patients receiving chemotherapy alone [32]. In the NRG/NSABP B-31 trial, where early HER2+ BC patients were randomized to receive either chemotherapy alone or in combination with trastuzumab, strTIL levels were reported to be positively associated with improved DFS in both trastuzumab-treated and trastuzumab-untreated patients, while no association between TIL levels and trastuzumab benefit was found [33].

In the Aphinity phase III trial, randomizing HER2+ BC patients to receive either pertuzumab or placebo in combination with standard adjuvant chemotherapy plus trastuzumab, TILs were associated with iDFS when considering the two arms pooled, thus corroborating the positive prognostic impact of TILs in patients receiving adjuvant chemotherapy plus anti-HER2 blockade [34].

TIL levels have also been assessed on 866 early BC samples from the randomized non-inferiority Shorther trial [35,36], where 1254 patients were randomly assigned to receive adjuvant anthracycline–taxane-based chemotherapy plus either 1 year or 9 weeks of trastuzumab. Higher TILs were found to be significantly, positively and independently associated with DDFS, with a 27% reduction in the risk of events for each 10% TILs increment. Moreover, an interaction between the magnitude of the TIL prognostic impact and trastuzumab duration was found. In detail, patients with low TILs (<20%) derived a significant benefit from trastuzumab administered for 1 year vs. 9 weeks (5-yy DDFS in short vs. long arms: 88.8% vs. 93.3%, respectively; HR 1.75, 95% CI 1.09–2.80; *p* = 0.021), while patients with higher TIL levels (>20%) experienced excellent prognosis in both arms, with numerically higher DDFS rates in patients treated with 9 weeks of trastuzumab (DDFS 97.6% and 93.7% for the short and long arm, respectively, HR 0.23, 95% CI 0.05–1.09, *p* = 0.064; *p* for interaction = 0.015). Intriguingly, these results suggest that TILs may have a role in identifying early HER2+ BC patients who might safely undergo a de-escalated anti-HER2 treatment in the adjuvant setting. Notably, by integrating 17 clinicopathological and genomic variables from a subset of patients enrolled in this trial, a multivariate prognostic tool—the so-called HER2DX model—was recently developed, encompassing, among others, TILs, PAM-50 intrinsic subtypes and 13 individual genes. Interestingly, HER2DX was found to be significantly associated with distant-MFS [37]. HER2DX was further validated for its prognostic role in an independent evaluation cohort of patients with early-stage HER2+ BC enrolled in four other clinical studies (conducted in either the adjuvant or neoadjuvant setting), thus emerging as a promising tool potentially capable of optimizing the prognostic stratification of patients with early HER2 BC [37]. The clinical relevance of TILs in HER2+ BC was also evaluated in the neoadjuvant setting. Interestingly, in a meta-analysis including five randomized clinical trials testing diverse anti-HER2-based treatments in the neoadjuvant setting, significantly increased pCR rates were observed in the high TIL subgroup (OR 2.46 (95% CI 1.36–4.43, *p* = 0.003), while no interaction was found between TIL levels and response to either chemotherapy or anti-HER2 treatments [38]. In addition, in the already mentioned pooled analysis of six randomized clinical trials by the German Breast Group - GBG (in all but two trials, HER2+ BC patients received anti-HER2 therapy as part of the neoadjuvant treatment), higher TILs were associated with both DFS and increased rates of pCR after neoadjuvant chemotherapy [8].

Overall, the interpretation of results regarding the clinical relevance of TIL evaluation in HER2+ disease may be complicated by the great heterogeneity of this BC subtype. Indeed, it has consistently been reported that among HER2+ tumors, all intrinsic molecular subtypes by PAM50 analysis can be detected, with non-HER2-enriched BC covering more than a half of all HER2+ tumors [39,40,41]. Indeed, this may be particularly relevant in the light of available evidence suggesting an association between TIL levels and molecular intrinsic subtypes, with HER2-enriched tumors showing the highest levels of immune infiltration [42,43]. In addition, in the abovementioned Shorther trial, the observed association between phosphatidylinositol-4,5-bisphosphate 3-kinase (PIK3CA) mutations and more favorable prognosis was hypothesized to be linked, at least in part, to immune infiltration, given the fact that PIK3CA-mutated tumors also harbored higher TILs as compared to PIK3CA wild-type tumors [44], thus adding a further layer of complexity on HER2+ BC heterogeneity.

##### Luminal-Like BC

As already mentioned, a large body of evidence suggests a more limited clinical relevance of TILs in luminal-like BC. Indeed, biomarker analyses from several clinical trials of diverse adjuvant chemotherapy combinations followed by endocrine therapy failed to report any association between TILs and prognosis in this BC subtype [30,45,46,47,48]. Recently, in a large retrospective case–cohort study including more than 980 early luminal-like BC patients, the evaluation of immune infiltrates revealed a significant association between higher TIL levels and unfavorable clinicopathologic features, including, among others, high ki67 levels, low ER expression and a luminal-B phenotype. In addition, although no significant association between TILs and outcome was observed in the overall cohort, a positive prognostic impact of TILs was revealed in luminal-B-like BC patients defined by the presence of high Ki67 levels (≥20%), while an inverse prognostic association was observed in patients with lower Ki67. Based on these results, it can be speculated that the inconclusive data on TIL clinical relevance in luminal-like BC may derive from the biological heterogeneity of this BC subtype [49]. In this context, luminal-A and luminal-B phenotypes may exhibit profound differences in terms of immunobiology, with substantial implications in terms of TIL biological and clinical relevance.

Moreover, higher TIL levels have been associated with increased pCR rates after neoadjuvant chemotherapy also in luminal-like BC; however, survival analysis from a large study pooling individual patient data from several neoadjuvant trials revealed a counterintuitive association between higher TILs and shorter OS in the luminal-like/HER2-negative BC subgroup [8]. Indeed, although these results may have mainly been driven and biased by the fact that the great majority of luminal-like/HER2-BC patients failed to achieve pCR, available evidence overall highlights the imperative need to further refine the prognostic value of immune infiltrates in this BC subtype, also taking into account the immune biological heterogeneity within luminal-like breast tumors. Interestingly it has been suggested that a more comprehensive evaluation of immune infiltrate composition may help better capture the role of immune factors in luminal-like BC [6].

Given the lack of conclusive data on the prognostic role of TILs in luminal-like/HER2-negative BC, the routine quantification of immune infiltrates is not recommended in this BC subtype.

#### 2.1.2. Clinical Relevance of Different TIL Subsets

##### T cells

The clinical relevance of T cells and their subsets has been suggested in both unselected BC and specific BC subtypes.

In detail, the prognostic value of different T cell subpopulations has been explored in large adjuvant cohorts. Cytotoxic CD8+ TILs have been positively associated with longer BC-specific survival (BCSS) in a cohort of 1334 unselected BC patients undergoing surgery for their primary disease [50]. In addition, in the neoadjuvant setting, several authors reported a positive significant association between pCR rates and high levels of total T cells (CD3+) [25,51,52,53,54], as well as high infiltration of T helper (CD4+) [55] subsets and cytotoxic (CD8+) [27,53,54,56,57] in multiple retrospective series of unselected BC patients receiving neoadjuvant chemotherapy (anthracycline, anthracycline+taxane or taxane). In addition, counterintuitively, total T cells have been negatively associated with DFS rates in unselected BC patients receiving anthracycline+taxane-based neoadjuvant chemotherapy [55].

In the TN subtype, higher total counts of CD8+ and CD4+ TILs, quantified by different methods, were significantly associated with favorable outcomes in large cohorts of early TNBC patients mostly treated with standard adjuvant therapy [50,56,57]. A further refinement of the relevance of characterizing TIL subsets in TNBC comes from a recent report, which identified four distinct TME subtypes (ID, immune-desert; MR, margin-restricted; SR, stroma-restricted; and FI, fully inflamed) by integrating gene expression signatures with spatial patterns of CD8+ T cell localization in intratumoral and stromal matched samples of treatment-naïve TNBC. In detail, both MR and ID tumors were characterized by low CD8+ infiltration, with the first showing CD8+ accumulation at the margins; in contrast, FI and SR tumors were characterized by abundance in CD8+ T cells, with SR tumors showing CD8+ infiltration restricted to the stroma, and FI showing CD8+ T cell infiltration in both stromal and epithelial compartments. Interestingly, metasignatures defining the four TME subtypes were found to be prognostic in an independent validation cohort of TNBC bulk samples, with patients with low CD8+ T cells infiltration (including either MR and ID TME subtypes) showing the poorest outcome, while those with CD8+ T cell abundance, including FI and SR TME subsets, experienced the most favorable prognosis [58]. In the neoadjuvant setting, high CD3+ TILs [59], as well as high CD8+ [59] and CD4+ subsets, have been positively associated with pCR after neoadjuvant chemotherapy (anthracycline+/−taxane) [60]. In addition, a high CD8/FOXP3 ratio has been positively associated with pCR and prognosis in patients treated with anthracycline+taxane-based neoadjuvant treatment. In addition, evaluation of the CD8/FOXP3 ratio on RD after neoadjuvant chemotherapy (anthracycline+/−taxane) has been suggested as potentially capable of identifying patients at lower risk of relapse and/or death despite the failure to achieve pCR, thus representing a promising tool for better stratifying prognosis of TNBC patients treated in the neoadjuvant setting and with RD at the time of surgery [61]. Interestingly, TIL content and TIL subsets were recently evaluated in a prospective cohort of primary and metastatic BC using multiparameter flow cytometry. T cells (CD3+) proved to be the predominant TIL population [12]. In addition, higher CD3+ concentrations were associated with a decreasing CD4/CD8 ratio, as well as increasing CD8+ T cells with features of tissue-resident memory (CD8+CD103+), thus possibly reflecting a more robust antitumor immune response. Notably, single-cell RNA sequencing revealed an association between a CD8+ tissue-resident memory signature and improved outcome in almost 330 primary early TNBC patients treated with standard chemotherapy, with a stronger prognostic impact as compared to CD3, CD8 or CD103 single-gene expression. Based on these promising results, it might be speculated that tissue-resident memory cells may be one of the key mediators of antitumor immunosurveillance, and, as such, they may represent the crucial determinant of the positive prognostic role of high TIL levels in early TNBC patients receiving standard treatments.

Furthermore, available evidence overall suggests that in TNBC, overall strTIL count, CD8 expression and FOXP3 expression are strongly correlated with each other. This observation supports the notion that the quantification of general TILs may reflect a general state of immune activation [13,62].

In a HER2+ BC subpopulation, high overall T cells (CD3+) and high cytotoxic T cells (CD8+) were positively associated with pCR rates after anthracycline+taxane-based neoadjuvant treatment, while a positive association between CD8+ TILs and pCR was described in HER2+ BC patients receiving anti-HER2-based neoadjuvant treatment. Interestingly, a high CD8/FOXP3 ratio was associated with both increased pCR and favorable prognosis in patients receiving neoadjuvant treatment with anthracycline+taxane-based chemotherapy and anti-HER2 agents [63,64].

Interestingly, an unsupervised clustering analysis of immune markers performed in the context of the TBCRC006 trial (neoadjuvant lapatinib plus trastuzumab without chemotherapy) revealed that the patient cluster characterized by high CD4+ strTILs, high CD8+ strTILs and high CD20+ intTILs was significantly, positively and independently associated with pCR after chemotherapy-sparing anti-HER2-based neoadjuvant treatment [65]. In the same study, similarly to TNBC, strTIL density assessed by H&E slides was reported to be correlated with CD8+ and CD4+ T cell subtypes (both in the stromal and intratumoral compartments) [65], thus strengthening the hypothesis that simple morphologic evaluation of immune infiltrates may represent an indicator of the antitumor adaptive immune response.

Luminal-like/HER2- BC studies investigating the clinical relevance of specific subsets of TILs reported promising results [66]. In particular, cytotoxic T cells (CD8+) were negatively associated with prognosis in a large adjuvant cohort including one randomized trial and three observational studies [49]. In addition, both CD8 +TILs and a high CD8/FOXP3 ratio have been associated with pCR rates in luminal-like BC patients with a luminal-B phenotype receiving neoadjuvant treatment with anthracycline (+/−taxane) [66,67]. Available data also suggest a role of specific TIL subsets in luminal-like BC patients receiving hormonal therapy. In particular, the presence of CD4+FOXP3+ TILs has been consistently associated with poor outcome in retrospective series of early luminal-like BC patients receiving endocrine treatment with selective estrogen receptor modulators (SERMs, such as tamoxifen), especially in the absence of concurrent CD8+ TIL infiltration [48,68]. In addition, in a large analysis including more than 500 HR+ BC patients randomized to receive adjuvant tamoxifen vs. no adjuvant treatment, CD8+ T cells were found to be significantly associated with decreased RFS rates in both tamoxifen-treated and untreated cohorts, regardless of HER2 status. Interestingly, a weak correlation between the phosphorylation levels of the phosphoinositide 3-kinases (PI3K) pathway and lymphocytic infiltration, especially for FOXP3+ cells, was observed, thus revealing a possible link between the PI3K pathway and immune infiltrate composition [69]. In contrast, no association between immune infiltrate subsets and survival rates in patients receiving aromatase inhibitors has been observed.

Although available evidence regarding the role of immune infiltrate composition in luminal-like BC may appear scattered, it may be speculated that the complex interplay between HR+ tumor cells, estrogen levels and inflammatory mediators, as well as the patient’s menopausal status and the type of endocrine agent (SERM vs. aromatase inhibitor), may contribute to unbalancing the immunological properties of regulatory T cells towards a more immunosuppressive or otherwise more antitumorigenic phenotype, in the context of the luminal-like BC-TME [13].

##### B Cells

Solid data on the clinical relevance of assessing B lymphocytes in BC are currently missing. However, preliminary evidence suggests an association of B cells with improved outcome in TN/basal-like early BC [70,71]. In addition, the presence of B lymphocytes (CD20+) has been associated with higher pCR rates after anthracycline–taxane-based neoadjuvant treatment [55,72], as well as after dual HER2 blockade (without chemotherapy) in HER2+ BC patients [65].

It should be noted that several other authors failed to report any association between B cells, T cells or their subsets with either prognosis or pCR rates after neoadjuvant treatment in early BC patients [61], thus precluding the possibility to draw definitive conclusion about the biological and clinical relevance of their assessment in early BC. In addition, the abovementioned studies adopted highly heterogeneous scoring systems and cutoffs for defining a positive staining, none of which standardized, thus further complicating the interpretation and generalization of available data. For these reasons, identification and quantification of specific immune cell subsets is currently not recommended in the routine pathology practice and should be restricted to the research setting.

#### 2.1.3. Immune Infiltrates in the Advanced Setting

In contrast to the early setting, the immune landscape of metastatic BC is broadly unexplored. As already mentioned, the metastatic TME appears to be colder as compared to the early setting, as highlighted by available evidence suggesting that BC metastases, as compared to matched primary tumors, show not only lower TIL levels [9,10,11,12,73] but also depleted immune functions, reflected by both the downregulation of immune-activating molecules and the upregulation of those with immunosuppressive properties, thus strengthening the hypothesis that metastatic BC may evade the immune surveillance by unbalancing the TME towards an inert phenotype [73].

Indeed, in the majority of studies investigating the clinical impact of immune aspects in advanced BC patients, immune infiltrates have been assessed mostly on primary tumor samples, thus precluding the possibility to draw definitive conclusions. However, an interesting insight comes from a recent retrospective study aiming at characterizing the immune infiltrates of BC metastases. In detail, secondary lesions from 94 advanced BC patients (43 TNBC and 51 HER2+BC) were evaluated in terms of morphologic TIL evaluation as well as specific subsets of immune cells. TIL levels did not significantly differ between TNBC and HER2+ BC samples [13]. Interestingly, lung metastases were confirmed to be generally more infiltrated by TILs as compared to other sites. In addition, the TME of skin lesions appeared unbalanced towards an immunosuppressive phenotype since a lower CD8/FOXP3 ratio was observed as compared to other metastatic sites. Survival analysis revealed a significant association between TILs and prognosis in both TNBC and HER2+ BC cohorts. However, while TNBC patients exhibiting higher TILs experienced better OS rates, counterintuitively, an association in the opposite direction was observed in the HER2+ BC subgroup. This latter finding appears in contrast to data coming from the translational analysis of the Cleopatra study, where more than 800 advanced HER2+ BC patients were randomized to receive first-line therapy with taxane-based chemotherapy + trastuzumab in combination with either pertuzumab or placebo. For each 10% increase in TIL levels, an improvement in OS rates was observed (HR 0.89 (95% CI 0.83–0.96), *p* = 0.0014), while no association between TILs and the magnitude of benefit from the addition of pertuzumab was reported [14]. A possible explanation for such inconsistency is that in the Cleopatra study, TILs were assessed almost entirely in primary BC samples, thus possibly failing to capture the actual role of TILs in HER2+ BC patients with advanced disease. In contrast, a secondary analysis of the MA.31 randomized clinical trial of paclitaxel plus either trastuzumab or lapatinib failed to report any prognostic or predictive role of the morphologic evaluation of TILs on H&E-stained slides. However, interestingly, when considering specific subsets of TILs, among patients treated in the lapatinib arm, those exhibiting lower CD8+ TIL levels experienced less favorable PFS and OS rates as compared to patients with higher CD8+ TILs [74].

Given the substantial inconsistency of these results, especially when considering advanced HER2+ BC, no definitive conclusions can be drawn on the clinical relevance of the evaluation of immune infiltrates in patients with advanced disease receiving chemotherapy and/or HER2 blockade.

#### 2.1.4. Role of Immune Infiltrates in the Era of Immunotherapy

At present, the only established predictive biomarker for immunotherapy efficacy in metastatic BC is represented by PD-L1 expression, as emerged in the context of randomized phase III trials for TNBC BC. The Impassion 130 trial evaluated the addition of atezolizumab to first-line chemotherapy with nab-paclitaxel in 902 metastatic TNBC patients, showing a significant PFS benefit in both the ITT population and the PD-L1-positive (PD-L1+) cohort, defined by the presence of at least 1% staining of PD-L1 (by SP142 assay) on immune cells. In addition, although OS was not significantly improved in the ITT, an increase in OS was observed among the PD-L1+ subgroup in the immunotherapy-containing arm (statistical significance was not formally tested due to the hierarchical plan of the study) [75]. In the KEYNOTE 355 trial comparing treatment of the physician’s choice (several chemotherapeutic agents allowed) in combination with either placebo or pembrolizumab for the first-line treatment of advanced TNBC, PD-L1 status was assessed by the combined positive score (CPS), which takes into account not only PD-L1 expression on immune cells but also on tumor cells. Results from the KEYNOTE 355 trial showed a significant PFS benefit with the addition of immunotherapy to first-line chemotherapy in the PD-L1+ population, defined by a CPS ≥ 10. Statistical significance was, however, not met when defining PD-L1+ patients by CPS ≥ 1 [76].

Based on these findings, the FDA granted accelerated approval to both atezolizumab and pembrolizumab in combination with chemotherapy for the first-line treatment of unresectable locally recurrent or metastatic TNBC patients whose tumors express PD-L1 (PD-L1 staining on immune cells ≥1% for the use of atezolizumab and CPS score ≥10 for the use of pembrolizumab), as determined by an FDA-approved test. Indeed, the FDA also approved the Ventana PD-L1 SP142 (Medical System Inc., Tucson, AZ, USA) and the PDL1 22C3 pharmDx (Dako North America, Inc., Carpinteria, CA, USA) assays as companion diagnostics for selecting advanced TNBC patients for atezolizumab and pembrolizumab, respectively.

The Impassion131 phase III trial, which compared first-line paclitaxel with either placebo or atezolizumab, failed to show a significant PFS improvement in the immunotherapy arm. Unexpectedly, PD-L1+ status (≥1% PD-L1 staining on immune cells with SP142 assay) did not influence atezolizumab benefit, with even a trend towards a detrimental effect in the preliminary OS analysis. Although hypothetical reasons for inconsistent results may rely on treatment-related factors such as steroid premedication or different backbone chemotherapies, these results may also suggest PD-L1 expression as a suboptimal predictive biomarker in this setting [76,77]. Thus, there is an impelling need to further deepen the biological role as well as the clinical relevance of PD-L1 expression in patients receiving immunotherapy in the advanced setting and to explore other predictors of immunotherapy efficacy [78].

In the early setting, randomized clinical trials comparing chemotherapy alone vs. the combination of chemotherapy plus immune checkpoint blockade as neoadjuvant treatment failed to show an association of PD-L1 expression with the magnitude of benefit from immunotherapy [79,80,81,82,83]. On the other hand, in this context, a relationship between PD-L1+ status at baseline and increased pCR rates after neoadjuvant treatment with chemotherapy or chemotherapy + immune checkpoint blockade has been consistently reported, as summarized in Table 1, thus suggesting that the expression of PD-L1 in early TNBC may be associated with a general state of increased treatment sensitivity rather than a specific predictive value towards immunotherapy efficacy.

In detail, in the GeparNuevo trial, where the addition of durvalumab to anthracycline–taxane neoadjuvant treatment for TNBC patients resulted in numerically (not statistically significant) higher pCR rates (delta pCR = 9.2%), PD-L1+ status was not predictive of durvalumab benefit. However, a trend towards increased pCR rates irrespective of the treatment arm was observed in PD-L1+ patients [80].

In the phase Ib KEYNOTE 173 trial, where pembrolizumab was associated with several chemotherapeutic regimens, pre-treatment PD-L1+ status was significantly and positively associated with increased pCR rates. However, the absence of a control arm precludes the possibility to establish a predictive role of PD-L1 status in terms of pembrolizumab benefit [83].

In the phase III Keynote 522 trial, where the addition of pembrolizumab to neoadjuvant anthracycline–taxane–carboplatin-based chemotherapy significantly improved pCR rates, PD-L1+ status was positively associated with increased pCR rates, with no predictive impact in terms of pembrolizumab benefit [79].

Similarly, in the Impassion 031 phase III trial, the addition of atezolizumab to anthracycline–taxane-based neoadjuvant chemotherapy resulted in a significant increase in pCR rates irrespective of PD-L1 status [81,82].

The NeoTRIPaPDL1 phase III trial failed to show any pCR improvement with the addition of atezolizumab to taxane–carboplatin-based neoadjuvant chemotherapy (pCR was a secondary endpoint). In the translational analysis, PD-L1 status was found to positively affect pCR rates in the overall cohort and in each treatment arm, with no predictive role in terms of atezolizumab benefit [84,85].

Overall, available data highlight the current lack of a reliable and comprehensive predictive biomarker capable of identifying patients who may likely benefit from immune checkpoint inhibitors. In this context, different settings (metastatic vs. neoadjuvant), different assays (% of PD-L1+ immune cells and/or tumor cells, CPS, semiquantitative scores), different immune checkpoint inhibitors (anti-PD-L1 vs. anti-PD1) and different chemotherapeutic partners may have played a role in the generation of such inconclusive and inconsistent data [68].

In this context, beyond PD-L1 expression, several other biomarkers proved to be potentially capable of improving our ability to select patients for immunotherapy. Focusing on BC immune infiltrates, interestingly, accumulating evidence suggests that the morphologic evaluation of TILs may have a role in this regard. Notably, it has been consistently reported that PD-L1 expression is predominant on immune cells rather than tumor cells [13,86]. In addition, as already mentioned, it has been reported that PD-L1 expression and TIL levels are strongly correlated with each other, especially in the TNBC subtype, thus suggesting that the simple morphological evaluation of TILs may serve as a surrogate reflecting a general state of immune activation [13,68].

Translational analyses of several trials testing diverse immune checkpoint blockade strategies suggested a potentially clinically relevant role of TILs in BC patients treated with immunotherapy either in the early or advanced setting, as summarized in Table 2. In the phase II KEYNOTE-086 study which included both heavily pre-treated advanced TNBC patients irrespective of PD-L1 expression (cohort A) and PD-L1+ (CPS ≥ 1%) TNBC patients not previously treated in the advanced setting (cohort B), strTILs were suggested as a promising tool potentially capable of identifying patients who are more likely to benefit from pembrolizumab as a monotherapy in terms of overall response rate (ORR) (combined cohorts: OR for ORR = 1.26 (95% CI 1.03–1.55), *p* = 0.01). Interestingly, although responders showed higher strTILs as compared to non-responders in both cohorts, the highest delta was observed in the first-line setting subgroup [87].

In the phase III KEYNOTE-119 trial, where single-agent pembrolizumab failed to improve OS as compared to chemotherapy per the physician’s choice in 622 pre-treated patients with advanced TNBC [88], strTILs as both continuous and categorical variables (cutoff 5%) were significantly associated with all clinical outcomes (best overall response—BOR, ORR, disease control rate—DCR, OS and PFS) only in the immunotherapy arm, thus suggesting a potential predictive role of TILs in predicting pembrolizumab benefit in heavily pre-treated advanced TNBC patients [89].

The biomarker analysis from the abovementioned Impassion 130 study revealed an enrichment for PD-L1+ immune cells in BC samples showing intermediate/high levels of TILs, by adopting 10% as the cutoff. In addition, although the predictive role of TILs (as a categorical variable) in terms of atezolizumab benefit seemed to be restricted to the subgroup of patients with concurrent PD-L1+ status, the largest magnitude of PFS improvement with the addition of atezolizumab to nab-paclitaxel was observed in the subgroup of TNBC patients exhibiting both intermediate/high TILs and PD-L1+ status [86].

In the neoadjuvant KEYNOTE-173 trial, higher levels of both baseline and on-treatment (after one dose of single-agent pembrolizumab) TILs were associated with increased pCR rates after neoadjuvant pembrolizumab+chemotherapy. In addition, a numerically higher increase in TILs from baseline to on-treatment samples was correlated with higher pCR rates. Although promising, no conclusion can be drawn on the possible predictive role of TILs in terms of response to neoadjuvant pembrolizumab given the lack of a pembrolizumab-free arm [83].

The immune biomarker correlative analysis of the NeoTRIPaPDL1 trial revealed an association between high levels of TILs (TILs ≥ 40%) and increased pCR rates in both atezolizumab and control arms. However, no interaction was found between TIL levels and the treatment arm [85].

Biomarker analyses of the GeparNuevo phase II trial revealed that higher strTILs were significantly and positively associated with pCR in the overall cohort but were not specifically predictive of durvalumab benefit, while intTILs were associated with neither pCR rates nor durvalumab benefit. However, notably, an increase in intTIL levels from baseline to the post-window samples (durvalumab monotherapy for 2 weeks before the start of chemotherapy) was reported to be predictive of the subsequent achievement of pCR, specifically in the durvalumab arm (statistical significance of the interaction test not formally met), thus suggesting that the migration of TILs from the peritumoral area into the tumor cell nests induced by durvalumab may be a potential predictor of the subsequent response to this immune checkpoint inhibitor [80].

The association between immune infiltrates and response to immunotherapy has also been investigated in the HER2+ BC subtype.

In the phase I-IIb PANACEA trial, advanced HER2+ BC patients were treated with pembrolizumab+trastuzumab. Interestingly, the biomarker analyses revealed a correlation between PD-L1 positivity and higher TIL levels. In addition, higher TIL levels were observed in responders as well as in patients showing disease control. Although the lack of a control arm precludes clearly stating the predictive value of TILs in terms of pembrolizumab benefit in HER2+ BC patients, translational results from the PANACEA study are encouraging and deserve further investigation [11].

Similarly, in the KATE2 trial, where 202 advanced HER2+ BC patients received the combination of either TDM1+placebo or TDM1+atezolizumab, a borderline significant association was found between higher TILs (≥5%) and increased benefit from the addition of immunotherapy to anti-HER2 treatment, thus strengthening the potential predictive role of TILs in terms of immune checkpoint blockade efficacy [90,91].

Although results from trials testing immunotherapy in luminal-like BC are overall modest, translational analyses allow capturing interesting insights into the TME of this BC subtype.

The translational analyses from the GIADA trial which enrolled 43 luminal-B-like early BC patients to receive, in the neoadjuvant setting, anthracycline-based induction chemotherapy followed by the combination of nivolumab plus endocrine therapy revealed significantly higher strTIL levels in patients achieving pCR. In addition, a chemotherapy-induced increase in TILs (from baseline to post-anthracycline samples) was also observed. A similar behavior was observed for activated cytotoxic T cells, while regulatory T cell dynamic changes from baseline to post-CT samples went in the opposite direction [92].

### 2.2. Tumor-Infiltrating Dendritic Cells (DCs)

DCs represent antigen-presenting cells and are crucial players of the adaptive immune system. Interestingly, an impairment of their function, mostly in terms of defective antigen presentation, has been observed in patients with cancer [93]. It has been suggested that DCs (S100+) in the TME show a compartmentalization pattern, with immature DCs (CD1a-) generally residing within the tumor and mature DCs (CD83+) typically adhering selectively to the peritumoral area [94]. In addition, the presence of DCs was reported to well correlate with other immune biomarkers such as general TILs and T-regulatory and T-cytotoxic subsets, as well as tertiary lymphoid structures [95].

Despite their well-recognized biological relevance, their role in BC is largely unexplored.

The presence of S100+ tumor-infiltrating DCs has been correlated to unfavorable clinicopathologic features, such as higher tumor grade, larger tumor size, nodal involvement and HR-negative status [96,97], while an inverse association between DCs and negative nodal status has been suggested when specifically distinguishing mature DCs (CD83+) [98].

Preliminary data also suggest a prognostic role of tumor-infiltrating DCs in BC patients. In detail, a retrospective analysis of 130 tumor samples from unselected early BC patients undergoing curative surgery showed a significant and independent positive relationship between higher mature DCs and longer RFS and OS [98]. Interestingly, the mature DC prognostic value was reported to be even larger when considering BC patients with nodal involvement. This observation was further confirmed in a large retrospective cohort of 681 TNBC patients, where the presence of DCs on pre-therapeutic tumor samples was found to be prognostic (in terms of RFS and OS) only in node-positive patients [96].

These data overall highlight a promising prognostic role of DCs in early BC patients. In particular, although preliminary, available evidence suggests that the presence of DCs may help to identify a subgroup of patients with good prognosis despite the presence of nodal involvement at baseline. For this reason, further research in this field is highly encouraged.

## 3. Innate Immunity

### 3.1. Tumor-Associated Macrophages (TAMs)

Although TAMs represent a highly heterogeneous immune cell population, they can be commonly dichotomized in two polarized phenotypes, M1 and M2, with the former traditionally associated with antitumor effects, and the latter typically showing protumorigenic characteristics. Surface markers of M1-polarized TAMs are, among others, the costimulatory molecules CD80 and CD86, while the expression on the TAM surface of CD163, CD204 and CD206 is typically associated with M2 polarization. CD68 is instead considered a pan-macrophage marker.

In recent decades, TAMs have gained increasing attention in the light of accumulating preclinical evidence suggesting their involvement in BC tumorigenesis, metastasis and resistance to treatments. In detail, an association between TAMs and protumorigenic processes has been reported, including the activation of oncogenic pathways, the induction of epithelial-to-mesenchymal transition and the promotion of tumor-associated neovascularization [99]. In addition, results from several studies on preclinical BC models suggest that TAMs recruitment into the BC-TME as well as their polarization into an M2 phenotype could hamper the activity of cytotoxic therapies, endocrine treatments, anti-HER2-targeted agents and even immune checkpoint blockade. Although preclinical evidence overall highlights that TAMs represent a biologically relevant player in the context of the BC-TME, results from clinical studies, deepening their role in BC patients, are scattered and broadly inconsistent.

The infiltration of TAMs in the BC-TME has been generally associated with unfavorable clinicopathologic features, such as higher tumor grade, vascular invasion and greater tumor burden (larger tumor size and lymph node involvement), as well as hormone receptor-negative, HER2+ and basal-like phenotypes [50,100,101]. Notably, the evaluation of tumor-associated macrophage activity reflected by the expression of the CD68 gene is shared by several clinically validated genomic assays, such as the 21-gene assay (Oncotype Dx^®^) and the 70-gene assay (Mammaprint^®^) [102]. However, when investigating the possible prognostic role of infiltrating TAMs—either when considered as unpolarized TAMs or as selected subpopulations—inconclusive results have been obtained.

In detail, TAMs have been retrospectively reported to predict unfavorable survival rates in terms of DFS, BCSS and OS in unselected BC [101,103], as well as in ER+ or TNBC subtypes [104,105]. Similarly, non-polarized TAMs have been suggested as an independent negative prognostic factor for worse cumulative survival in two large retrospective studies, adopting a computational approach (CIBERSORT) for the analysis of more than 18,000 early BC samples [106,107]. However, an association in the opposite was shown in a large retrospective analysis (n = 478) of early BC patients, where increasing levels of TAMs (CD68+) were positively and independently associated with both DFS and BCSS. In addition, it should be noted that several other studies failed to report any association between unselected TAMs (CD68+) and prognosis [108,109].

As far as M2-polarized (CD163+) TAMs are concerned, results from several retrospective series support their negative prognostic role in unselected early BC patients as well as in TNBC and HER2+ subgroups [101,109,110,111]. Interestingly, the negative association between M2-TAMs and prognosis was further confirmed in a large series of almost 11,000 primary tumor samples, where the authors, by applying a CIBERSORT approach, found an association between M2-TAMs and worse cumulative survival in ER-BC patients [107]. These results appear consistent with the notion that M2 polarization of TAMs may unbalance the BC-TME equilibrium by establishing an immunosuppressive milieu, thus facilitating tumor immune escape.

Nevertheless, a prognostic association in the opposite direction has also been suggested in ER-BC patients, thus further highlighting the need for a more in-depth evaluation of the TAM prognostic value [112].

TAMs’ role in early BC patients has also been deepened regarding the potential association with systemic treatment response.

In detail, both increasing M2- and M1-polarized TAMs have been found to be positively associated with pCR rates in several series of unselected locally advanced BC patients receiving neoadjuvant chemotherapy [106,111]. In contrast, the gene expression profiling of more than 11,000 early BC tumor samples (CIBERSORT approach) suggested that baseline M2-polarized TAMs were capable of predicting the subsequent failure to achieve pCR after neoadjuvant chemotherapy. On the other hand, several other authors failed to report a significant association between baseline TAMs and pCR. In particular, in the translational analysis of 150 baseline tumor samples from a phase II trial of anthracycline–taxane-based treatment (+ bevacizumab), no significant association between M2-TAMs (CD163+) and pCR rates was reported [113].

It is currently unknown whether the inconsistency and inconclusiveness of results on the possible prognostic and/or predictive role of TAMs reflect an actual lack of biological/clinical relevance in BC or are rather the result of heterogeneity in TAM assessment in terms of assays and cutoff points for classifying high TAM infiltration, as well as TME compartments for TAM inclusion. In addition, the majority of the above-mentioned studies adopted CD68 as a pan-macrophage marker. However, it should be noted that a wide range of other immune cell populations, including granulocytes, fibroblasts, lymphocytes and dendritic cells, may also express CD68 on their surface, thus further complicating the interpretation of these results [96].

From a therapeutic point of view, several strategies adopting TAMs as a target have been or are currently being investigated, encompassing inhibition of TAM recruitment, promotion of TAM killing, modulation of TAM polarization and reduction in protumorigenic products released from TAMs. However, although preliminary, available results from pivotal trials testing anti-TAM-targeted strategies overall suggest a limited clinical activity/efficacy in BC patients. Ongoing trials currently investigating therapeutic strategies adopting TAMs or their function as a target are summarized in Table 3.

### 3.2. Natural Killer (NK) Cells

NK cells are cytotoxic members of the innate immune system, representing a crucial mediator of immunosurveillance and elimination in the cancer immunoediting process. However, their role in affecting BC prognosis and/or response to treatment has been poorly investigated. Preliminary data suggest that increased expression of NK-activating genes was associated with better RFS in a small series of unselected early BC patients [114]. In addition, in a small retrospective cohort of BC patients with locally advanced disease undergoing neoadjuvant treatment [115], higher levels of NK cells (CD56+) on pre-therapeutic samples were significantly associated with better pCR rates. The involvement of NKs in the ADCC-mediated mechanism of action of anti-HER2 monoclonal antibodies is well recognized. Interestingly, several strategies aiming at enhancing NK cell effector function (via ADCC) by anti-HER2 agents are currently under investigation and results are awaited [116].

Although promising, available evidence is immature and further efforts should be made in order to deepen the role of NKs in BC biology and clinical behavior.

## 4. Tertiary Lymphoid Structures (TLS)

TLS represent lymph node-like aggregates (with a germinal well-defined B lymphocyte- and follicular DC-rich area surrounded by a parafollicular T lymphocyte-rich area) which can be found in non-lymphoid organs, including cancer.

So far, data on the clinical relevance of TLS in BC remain speculative. However, in various retrospective series of cancer patients including breast tumors, the presence of TLS—along with general TIL assessment—has been associated with improved prognosis and increased pCR rates after neoadjuvant therapy in unselected BC as well as in patients with HER2+BC or TNBC subtypes receiving standard treatments. In addition, a recent retrospective study evaluating TLS in primary BC samples and paired metastases showed lower TLS in metastatic samples as compared to paired primary tumors, thus further confirming that BC metastases are generally characterized by lower immune infiltration as compared to primary BC [117].

## 5. Conclusions

In recent decades, the role of immune infiltrates in affecting both prognosis and response to systemic treatments in BC patients has become increasingly evident, especially in the early setting, as summarized in Table 4. The most exciting data concern the morphologic evaluation of TILs, which have been demonstrated to be strongly prognostic in TNBC and early HER2+ BC patients treated with standard systemic therapy either in the adjuvant or neoadjuvant setting, as well as being predictive for the subsequent achievement of pCR in patients receiving standard neoadjuvant treatment in all BC subtypes. Based on these solid data, TILs have recently reached level 1b evidence for their clinical validity in early TNBC, where their routine quantification and reporting as a prognostic biomarker are currently recommended.

In this context, given the growing pressure on pathologists to provide a reliable TIL quantification, a joint standardization effort by the International Immuno-oncology Biomarkers Working Group, gathering pathologists and oncologists from all over the world, resulted in the development of recommendations for TIL assessment on H&E-stained BC samples (www.tilsinbreastcancer.org). However, with these standardization efforts, the interobserver variability in TIL assessment still represents an issue, thus potentially undermining the reliability of the visual quantification of TILs in the real-world clinical practice. In this context, the incorporation of standard TIL assessment with machine learning platforms has been suggested as a strategic approach to further refine the reliability and reproducibility of TIL evaluation on a large scale. Indeed, this approach would improve the quantification accuracy, as well as the understanding of more complex spatio-morphological patterns, thus potentially allowing for providing standardized metrics in view of a rigorous validation. Moreover, given the increasing relevance of transcriptomic data in the context of BC immune-oncology, machine learning deconvolution approaches would assist pathologists in the characterization of immune infiltrates from molecular profiles [118].

In the metastatic setting, routine evaluation of PD-L1 expression has recently been endorsed by the FDA in order to identify previously untreated advanced TNBC patients suitable for a first-line treatment with chemotherapy plus the immune checkpoint inhibitor atezolizumab or pembrolizumab, following the results of the Impassion 130 and Keynote 355 randomized phase III trials, respectively. In this context, the evaluation of PD-L1 expression by the Ventana (SP142) and the Dako PharmDx (22C3) assays represents the only companion diagnostic devices currently endorsed by the FDA for identifying PD-L1+ TNBC patients suitable for atezolizumab and pembrolizumab, respectively. However, the predictive role of PD-L1 should still be considered suboptimal. Indeed, the contradictory results on the predictive value of PD-L1 expression observed in the Impassion 130 and Impassion 131 trials, which adopted the same PD-L1 assay (Ventana SP142) and evaluated the same immune checkpoint inhibitor (atezolizumab) in similar TNBC patient populations, highlight the pressing need to develop alternative or complementary immune predictive biomarkers—to be integrated with PD-L1 status—that are possibly easy, reliable and cost-effective to evaluate and implement. Based on available evidence, TILs may play a crucial role in this context. Interestingly, TILs proved to be strongly correlated with PD-L1 status. Indeed, it has been shown that the subgroup of patients exhibiting strTIL levels >20% was almost entirely PD-L1+ by the Ventana SP142 assay [119]. In this regard, the integrated evaluation of TILs and PD-L1 has not been deeply explored and it could represent a strategical tool potentially capable of refining our ability to select patients for immunotherapy and/or to prioritize samples for PD-L1 evaluation. Besides TILs and PD-L1 expression, several other components of BC immune infiltrates have been investigated as potential clinically relevant biomarkers, including the evaluation of specific TIL subsets, TLS and other key players of both the innate and adaptive immune responses, such as NKs, TAMs and DCs. Although promising, none of them are currently ready for prime time. In this context, further effort is necessarily needed in order to provide solid data supporting their analytical validity and, ultimately, their possible clinical validity and utility.

## Figures and Tables

**Table 1:** Summary of available evidence on PD-L1 evaluation in clinical trials testing immunotherapy in triple-negative breast cancer (TNBC) in the neoadjuvant setting.

Trial, Design	Population, PD-L1 Status (Assay)	Treatment Arms (n per arm)	pcR Rates		Biomarker Analysis
			% (95% CI)	OR (95% CI), *p*	
GeparNuevo [80], Phase II R	TNBC Any PD-L1 (on both IC and TC, by Ventana SP163)	(Window cohort) ^a^ → Durvalumab + NabP → Durvalumab + EC (88)	53.4 (42.5–61.4) Window cohort: 61.0	1.45 (0.80–2.63), *p* = 0.224 Window cohort: 2.22 (1.06–4.64), *p* = 0.035	Trend towards an association between PD-L1 and pCR, not predictive of durvalumab benefit
		(Window cohort) ^a^ → Placebo + NabP → Placebo + EC (86)	44.2 (33.5–55.3) Window cohort: 41.4		
Keynote-173 [83], Phase Ib	TNBC Any PD-L1 (CPS, by PharmDx 22C3)	Pembrolizumab + 6 CT regimens ^b^ (60)	Overall: 60 (range: 49–71)	NA	Significant association between PD-L1 and pCR
Keynote-522 [79], Phase III R	TNBC Any PD-L1 (CPS, by PharmDx 22C3)	Pembrolizumab + NabP–Carboplatin → A/E-C + Pembrolizumab (401)	64.8 (59.9–69.5)	Estimated treatment difference: 13.6% (5.4–21.8, *p* > 0.001)	Significant association between PD-L1 and pCR, not predictive of pembrolizumab benefit
		Placebo + NabP–Carboplatin → A/E-C + Placebo (201)	51.2 (44.1–58.3)		
Impassion-031 [81,82], Phase III R	TNBC Any PD-L1 (on IC, by Ventana SP-142)	Atezolizumab + NabP → Atezolizumab + AC (165)	58 (50–65)	Rate difference: 17%, (6–27, *p* = 0.0044)	Association between PD-L1 and pCR, not predictive of atezolizumab benefit
		Placebo + NabP → Placebo + AC (168)	41 (34–49)		
NeoTRIPaPDL1 [84], phase III R	TNBC Any PD-L1 (on IC, by Ventana SP142)	Atezolizumab + NabP–Carboplatin (138)	43.5 (35.1–52.2)	1.11 (0.69–1.79), *p* = 0.66	Significant association between PD-L1 and pCR (secondary endpoint), not predictive of atezolizumab benefit
		NabP–Carboplatin (142)	40.8 (32.7–49.4)		

Abbreviations: R, randomized; pCR, pathologic complete response, OR, odds ratio; TN, triple-negative; R, randomized; IC, immune cells, TC, tumor cells; NabP, nab-paclitaxel; EC, epirubicin–cyclophosphamide; HER2-, HER2-negative; NA, not available; *p*, paclitaxel; AC, doxorubicin–cyclophosphamide; CPS; combined positive score. ^a^ The window phase consisted of 2 weeks of either durvalumab or placebo single agent; it was stopped after 117 patients were recruited. ^b^ Six different CT regimens consisting of taxane (with or without carboplatin in various schedules)–anthracyclines+cyclophosphamide.

**Table 2:** Summary of phase II/III clinical trials investigating immunotherapy in BC with results from TIL analysis available.

Trial (Design)	Setting, BC Subtype	Treatment Arms	TIL Variable (Cutoff ^a^)	Outcomes	Results ^b^
Keynote-086 [87] (II) ^e^	Advanced, TNBC	Pembrolizumab (2 cohorts)	Binary (median)	ORR DCR	Combined cohort: OR for ORR = 1.26 (1.03–1.55), *p* = 0.01; OR for CDR 1.22 (1.02–1.46), *p* = 0.01
Impassion-130 [86] (III)	Advanced, TNBC	NabP–Atezo NabP–Pbo	Binary (10%)	PFS OS	Predictive for Atezo benefit only in PD-L1+: PD-L1+/strTILs low→ PFS: HR 0.74 (0.54–1.03), *p* = 0.07, OS: HR 0.65 (0.41–1.02) *p* = 0.06. PD-L1+/strTILs high → PFS: HR 0.53 (0.38–0.74), *p* < 0.005; OS: HR 0.57 (0.35–0.92), *p* = 0.02. PD-L1-/strTILs high → PFS: HR 0.99 (0.62–1.57), *p* = 0.97; OS: HR 1.53 (0.76–3.08), *p* = 0.24
Keynote-119 (III) [89]	Advanced, TNBC	Pembro CT PFC ^c^	Continuous, Binary (5%)	BOR DCR PFS OS	Positive association with clinical outcomes only in Pembro arm (*p* < 0.05) TILs <5% → Pembro vs. CT: median OS 5.9 vs. 8.8 months, HR 1.50 (1.14–1.97) TILs ≥5% → Pembro vs. CT: median OS 12.5 vs. 11.3 months, HR 0.75 (0.59–0.96)
Panacea [11] (Ib-II)	Advanced, HER2+	Trastuzumab–Pembro	Continuous	OR DCR	Positive association with ORR (*p* = 0.006) and DCR (*p* = 0.0006)
KATE-2 (II-R) [90,91]	Advanced, HER2+	TDM1–Atezo TDM1–Pbo	Binary (5%)	PFS OS	Borderline positive association with OS in the atezolizumab arm TILs < 5% → Atezo vs. Placebo: mPFS 5.5 (3.9-9.6) vs NE (4.0-NE), HR 0.62 (0.37-1.03). 1-year OS 84.2% vs. 100%, HR 1.43 (0.51–4.01) TILs ≥ 5% → Atezo vs. Placebo: mPFS 8.5 (6.2-NE) vs 5.3 (4.0-9.5). 1-year OS 90.8% vs. 83.5%, HR 0.55 (0.26–1.12)
GeparNuevo [80] (II R)	Neoadjuvant, TNBC	Durva ^d^→ Durva–NabP→ Durva–EC Pbo→ Pbo–NabP→ Pbo–EC	Continuous Categorical (10%, and 60%)	pCR	strTILs (continuous): positive association with pCR, no predictive for Durva benefit OR 1.23 (1.04–1.6), *p* = 0.019 in Durva arm; OR = 1.39 (1.12–1.74), *p* = 0.003 in Placebo arm. intTILs: increase between baseline and end of the window phase associated with Durva benefit. OR 9.36 (1.26–69.65) *p* = 0.029
NeoTRIPaPDL1 [85] (III)	Neoadjuvant, TNBC	Cb–NabP–Atezo Cb–NabP	Binary (40%)	pCR	Positive association with pCR, no predictive role Atezo arm: strTILs ≥40% vs. <40% → pCR 71.43% vs. 28.07%, *p* = 0.001 CT arm: strTILs ≥40% vs. <40% → pCR 63.16% vs. 33.9%, *p* = 0.009 No interaction with treatment arm.
GIADA [92] (II)	Neoadjuvant, Luminal-B ^e^	EC→ Nivo–ET	Continuous	pCR	Positive association with pCR (*p* < 0.001)

^a^ When applicable; ^b^ 95% confidence intervals reported in parentheses; ^c^ single agent chemotherapy per physician’s choice: capecitabine, eribulin, gemcitabine, vinorelbine; ^d^ in a proportion of patients, durvalumab was administered as induction treatment in the window phase. ^e^ luminal-B = hormone receptor-positive/HER2-negative, Ki67 ≥ 20% and/or grade 3. Abbreviations: BC, breast cancer; TN, triple-negative; str/int-TILs, stromal/intratumoral tumor-infiltrating lymphocytes; ORR, overall response rate; DCR, disease control rate; OR, odds ratio; CI, confidence interval; R, randomized; HR, hazard ratio; pCR, pathologic complete response, BOR, best overall response; (m)PFS, (median) progression-free survival; OS, overall survival; DCR, disease control rate; CT, chemotherapy; NabP, nab-paclitaxel; Pbo, placebo; Pembro, pembrolizumab; Atezo, atezolizumab; Cb, carboplatin; Durva, durvalumab; EC, epirubicin–cyclophosphamide; Nivo, nivolumab; ET, endocrine therapy.

**Table 3:** Summary of strategies targeting TAMs which are currently under investigation in breast cancer preclinical and clinical studies.

Mechanism of Action	Target	Drug	Partners	Trials
TAM killing	Membrane cell receptor activation	Trabectidine	CT, PARP inhibitor	NCT00050427 NCT00580112 NCT03127215
	CSF1-CSF1R inhibition	Emactuzumab Pexidartinib Lacnotuzumab Cabiralizumab	CT, immune checkpoint inhibitors	NCT02323191 NCT02760797 NCT01494688 NCT01596751 NCT01525602 NCT01042379 NCT02435680 NCT02807844 NCT03285607 NCT04331067
Inhibition of TAM recruitment	CCL2-CCR2 inhibition	Carlutumab	CT	NCT01204996
Modulation of TAM polarization (from M2 into M1 TAM phenotype)	Macrophage	Zoledronic Acid	NA	Approved in both early and metastatic settings
	CD40 stimulation	Selicrelumab	Other anti-TAM agents	NCT02225002 NCT02157831 NCT02665416 NCT02760797
	CR3 stimulation	1,3–1,6 β-glucan	Immunotherapy	NCT02981303
Induction of cancer cell phagocytosis by TAMs	CD47-SIRPα inhibition	TTI-621, ALX148 Hu5F9-G4	Immunotherapy, anti-VEGF agents, anti-HER2 agents	NCT02890368 NCT03013218 NCT02216409 NCT02953782
Regulation of antigen presentation by TAMs	TLR7 stimulation	Imiquimod 852A	CT, RT	NCT00899574, NCT01421017, NCT00821964, NCT00319748
Vaccination	NA	SV-BR-1-GM H2NVAC	Immunotherapy	NCT04418219, NCT04144023

Abbreviations: TAMs, tumor-associated macrophages; CT, chemotherapy; PARP, poly-ADP polymerase; M2pep, M2peptide; CSF1 (R), colony stimulating factor 1 (receptor); CCL2, chemokine C-C motif ligand 2; CCR2, CCL2 receptor; mAb, monoclonal antibody; Fbln7, fibulin 7; CXCL1, C-X-C Motif Chemokine Ligand 1 Pathway; ZnPPIX, zinc protoporphyrin 9; SIRPα, signal regulatory protein alpha; TLR7, toll-like receptor 7; GM-CSF, granulocyte-macrophage colony stimulating factor.

**Table 4:** Summary of clinical validity of TILs as a prognostic marker and potential clinical utility in patients with early breast cancer.

BC Subtype	Clinical Validity (Prognostic)	LoE	Evaluation Endorsed by Guidelines	Potential Clinical Utility ^b^ (to be Demonstrated)
TNBC	High TILs are associated with improved outcome [19,21,22,23,31,33,34,35,36]. High TILs are associated with increased pCR rate after neoadjuvant chemotherapy [8,39]. In TNBC, high TILs on residual disease after neoadjuvant chemotherapy are associated with improved outcome [24,27].	IB ^a^	Yes: Expert Opinion at the 16th St Gallen International Breast Cancer Conference [15]; ESMO 2019 Early Breast Cancer guidelines [16]; WHO classification of Tumors, Breast Tumors, 5th edition;	Integration with other clinicopathological variables to guide treatment de-escalation in low-risk patients (i.e., no anthracyclines or even no treatment; prognostic tool available at www.tilsinbreastcancer.org). Risk stratification in post-neoadjuvant setting based on TILs and RCB to guide the decision of further adjuvant treatment. Stratification factor in clinical trials.
HER2+ BC			Yes: ESMO 2019 Early Breast Cancer guidelines [16]; WHO classification of Tumors, Breast Tumors, 5th edition.	Integration in multiparametric scores to guide treatment escalation and de-escalation (i.e., HER2DX). Stratification factor in clinical trials.
HR+/HER2-BC	Not demonstrated: conflicting results from studies in the adjuvant setting; high TILs associated with increased pCR rate after neoadjuvant chemotherapy, but less favorable survival [8].	-	No	Unknown

^a^ Availability of reproducible results in archived tissues from independent randomized trials, conducted according to REMARK guidelines; ^b^ likelihood of improved outcomes when using TIL as a biomarker. Abbreviations: BC, breast cancer; LoE, level of evidence; TN, triple-negative; HR+, hormone receptor-positive; TILs, tumor-infiltrating lymphocytes; pCR, pathologic complete response; RCB, residual cancer burden.

## References

[1] Burugu S., Asleh-Aburaya K., Nielsen T.O. (2017). Immune infiltrates in the breast cancer microenvironment: Detection, characterization and clinical implication. Breast Cancer.

[2] Karn T., Jiang T., Hatzis C., Sanger N., El-Balat A., Rody A., Holtrich U., Becker S., Bianchini G., Pusztai L. (2017). Association Between Genomic Metrics and Immune Infiltration in Triple-Negative Breast Cancer. JAMA Oncol..

[3] Savas P., Caramia F., Teo Z.L., Loi S. (2014). Oncogene addiction and immunity: Clinical implications of tumour infiltrating lymphocytes in breast cancers overexpressing the HER2/neu oncogene. Curr. Opin. Oncol..

[4] Bianchini G., Gianni L. (2014). The immune system and response to HER2-targeted treatment in breast cancer. Lancet Oncol..

[5] Luen S., Virassamy B., Savas P., Salgado R., Loi S. (2016). The genomic landscape of breast cancer and its interaction with host immunity. Breast.

[6] Dieci M.V., Griguolo G., Miglietta F., Guarneri V. (2016). The immune system and hormone-receptor positive breast cancer: Is it really a dead end?. Cancer Treat. Rev..

[7] Stanton S.E., Adams S., Disis M.L. (2016). Variation in the Incidence and Magnitude of Tumor-Infiltrating Lymphocytes in Breast Cancer Subtypes: A Systematic Review. JAMA Oncol..

[8] Denkert C., von Minckwitz G., Darb-Esfahani S., Lederer B., Heppner B.I., Weber K.E., Budczies J., Huober J., Klauschen F., Furlanetto J. (2018). Tumour-infiltrating lymphocytes and prognosis in different subtypes of breast cancer: A pooled analysis of 3771 patients treated with neoadjuvant therapy. Lancet Oncol..

[9] Adams S., Schmid P., Rugo H.S., Winer E.P., Loirat D., Awada A., Cescon D.W., Iwata H., Campone M., Nanda R. (2019). Pembrolizumab monotherapy for previously treated metastatic triple-negative breast cancer: Cohort A of the phase II KEYNOTE-086 study. Ann. Oncol..

[10] Adams S., Loi S., Toppmeyer D., Cescon D., De Laurentis M., Nanda R., Winer E., Mukai H., Tamura K., Armtrong A. Phase 2 study of pembrolizumab as first-line therapy for PD-L1-positive metastatic triple-neative breast cancer: Preliminary data from Keynote-086 cohort B. Presented at the ASCO 2017.

[11] Loi S., Giobbie-Hurder A., Gombos A., Bachelot T., Hui R., Curigliano G., Campone M., Biganzoli L., Bonnefoi H., Jerusalem G. (2019). Pembrolizumab plus trastuzumab in trastuzumab-resistant, advanced, HER2-positive breast cancer (PANACEA): A single-arm, multicentre, phase 1b-2 trial. Lancet Oncol..

[12] Savas P., Virassamy B., Ye C., Salim A., Mintoff C.P., Caramia F., Salgado R., Byrne D.J., Teo Z.L., Dushyanthen S. (2018). Single-cell profiling of breast cancer T cells reveals a tissue-resident memory subset associated with improved prognosis. Nat. Med..

[13] Dieci M.V., Tsvetkova V., Orvieto E., Piacentini F., Ficarra G., Griguolo G., Miglietta F., Giarratano T., Omarini C., Bonaguro S. (2018). Immune characterization of breast cancer metastases: Prognostic implications. Breast Cancer Res..

[14] Luen S.J., Salgado R., Fox S., Savas P., Eng-Wong J., Clark E., Kiermaier A., Swain S.M., Baselga J., Michiels S. (2017). Tumour-infiltrating lymphocytes in advanced HER2-positive breast cancer treated with pertuzumab or placebo in addition to trastuzumab and docetaxel: A retrospective analysis of the CLEOPATRA study. Lancet Oncol..

[15] Burstein H.J., Curigliano G., Loibl S., Dubsky P., Gnant M., Poortmans P., Colleoni M., Denkert C., Piccart-Gebhart M., Regan M. (2019). Estimating the benefits of therapy for early-stage breast cancer: The St. Gallen International Consensus Guidelines for the primary therapy of early breast cancer 2019. Ann. Oncol..

[16] Loi S. (2019). The ESMO clinical practise guidelines for early breast cancer: Diagnosis, treatment and follow-up: On the winding road to personalized medicine. Ann. Oncol..

[17] Denkert C., Wienert S., Poterie A., Loibl S., Budczies J., Badve S., Bago-Horvath Z., Bane A., Bedri S., Brock J. (2016). Standardized evaluation of tumor-infiltrating lymphocytes in breast cancer: Results of the ring studies of the international immuno-oncology biomarker working group. Mod. Pathol..

[18] Dieci M.V., Radosevic-Robin N., Fineberg S., van den Eynden G., Ternes N., Penault-Llorca F., Pruneri G., D’Alfonso T.M., Demaria S., Castaneda C. (2018). International Immuno-Oncology Biomarker Working Group on Breast Cancer Update on tumor-infiltrating lymphocytes (TILs) in breast cancer, including recommendations to assess TILs in residual disease after neoadjuvant therapy and in carcinoma in situ: A report of the International Immuno-Oncology Biomarker Working Group on Breast Cancer. Semin. Cancer Biol..

[19] Loi S., Drubay D., Adams S., Pruneri G., Francis P.A., Lacroix-Triki M., Joensuu H., Dieci M.V., Badve S., Demaria S. (2019). Tumor-Infiltrating Lymphocytes and Prognosis: A Pooled Individual Patient Analysis of Early-Stage Triple-Negative Breast Cancers. J. Clin. Oncol..

[20] Salgado R., Denkert C., Demaria S., Sirtaine N., Klauschen F., Pruneri G., Wienert S., Van den Eynden G., Baehner F.L., Penault-Llorca F. (2015). The evaluation of tumor-infiltrating lymphocytes (TILs) in breast cancer: Recommendations by an International TILs Working Group 2014. Ann. Oncol..

[21] Leon-Ferre R.A., Polley M.Y., Liu H., Gilbert J.A., Cafourek V., Hillman D.W., Elkhanany A., Akinhanmi M., Lilyquist J., Thomas A. (2018). Impact of histopathology, tumor-infiltrating lymphocytes, and adjuvant chemotherapy on prognosis of triple-negative breast cancer. Breast Cancer Res. Treat..

[22] Park J.H., Jonas S.F., Bataillon G., Criscitiello C., Salgado R., Loi S., Viale G., Lee H.J., Dieci M.V., Kim S.B. (2019). Prognostic value of tumor-infiltrating lymphocytes in patients with early-stage triple-negative breast cancers (TNBC) who did not receive adjuvant chemotherapy. Ann. Oncol..

[23] de Jong V., Wang Y., Opdam M., ter Hoeve N., Jozwiak K., Hauptmann M., Stathonikos N., Horlings H., Broeks A., Michiels S. Prognostic value of tumor infiltrating lymphocytes in young triple-negative breast cancer patients who did not receive adjuvant systemic treatment. Presented at ESMO, 2020 Virtual Meeting.

[24] Dieci M.V., Criscitiello C., Goubar A., Viale G., Conte P., Guarneri V., Ficarra G., Mathieu M.C., Delaloge S., Curigliano G. (2014). Prognostic value of tumor-infiltrating lymphocytes on residual disease after primary chemotherapy for triple-negative breast cancer: A retrospective multicenter study. Ann. Oncol..

[25] Demaria S., Volm M.D., Shapiro R.L., Yee H.T., Oratz R., Formenti S.C., Muggia F., Symmans W.F. (2001). Development of tumor-infiltrating lymphocytes in breast cancer after neoadjuvant paclitaxel chemotherapy. Clin. Cancer Res..

[26] Ladoire S., Arnould L., Apetoh L., Coudert B., Martin F., Chauffert B., Fumoleau P., Ghiringhelli F. (2008). Pathologic complete response to neoadjuvant chemotherapy of breast carcinoma is associated with the disappearance of tumor-infiltrating foxp3+ regulatory T cells. Clin. Cancer Res..

[27] Luen S.L., Salgado R., Loi S. (2019). Residual disease and immune infiltration as a new surrogate endpoint for TNBC post neoadjuvant chemotherapy. Oncotarget.

[28] Prowell T.M., Beaver J.A., Pazdur R. (2019). Residual Disease after Neoadjuvant Therapy—Developing Drugs for High-Risk Early Breast Cancer. N. Engl. J. Med..

[29] Griguolo G., Pascual T., Dieci M.V., Guarneri V., Prat A. (2019). Interaction of host immunity with HER2-targeted treatment and tumor heterogeneity in HER2-positive breast cancer. J. Immunother. Cancer.

[30] Loi S., Michiels S., Salgado R., Sirtaine N., Jose V., Fumagalli D., Kellokumpu-Lehtinen P.L., Bono P., Kataja V., Desmedt C. (2014). Tumor infiltrating lymphocytes are prognostic in triple negative breast cancer and predictive for trastuzumab benefit in early breast cancer: Results from the FinHER trial. Ann. Oncol..

[31] Perez E.A., Ballman K.V., Tenner K.S., Thompson E.A., Badve S.S., Bailey H., Baehner F.L. (2016). Association of Stromal Tumor-Infiltrating Lymphocytes With Recurrence-Free Survival in the N9831 Adjuvant Trial in Patients With Early-Stage HER2-Positive Breast Cancer. JAMA Oncol..

[32] Perez E.A., Thompson E.A., Ballman K.V., Anderson S.K., Asmann Y.W., Kalari K.R., Eckel-Passow J.E., Dueck A.C., Tenner K.S., Jen J. (2015). Genomic analysis reveals that immune function genes are strongly linked to clinical outcome in the North Central Cancer Treatment Group n9831 Adjuvant Trastuzumab Trial. J. Clin. Oncol..

[33] Kim R.S., Song N., Gavin P.G., Salgado R., Bandos H., Kos Z., Floris G., Eynden G.G.G.M.V.D., Badve S., Demaria S. (2019). Stromal Tumor-infiltrating Lymphocytes in NRG Oncology/NSABP B-31 Adjuvant Trial for Early-Stage HER2-Positive Breast Cancer. J. Natl. Cancer Inst..

[34] Krop I., Paulson J., Campbell C., Kiermaier A., Andre F., Fumagalli D., de Haas S., Salgado R., Denkert C., Loibl S. (2019). Genomic correlates of response to adjuvant trastuzumab and pertuzumab in HER2 positive breast cancer: Biomarker analysis of the Aphinity trial. J. Clin. Oncol..

[35] Dieci M.V., Conte P., Bisagni G., Brandes A.A., Frassoldati A., Cavanna L., Musolino A., Giotta F., Rimanti A., Garrone O. (2019). Association of tumor-infiltrating lymphocytes with distant disease-free survival in the ShortHER randomized adjuvant trial for patients with early HER2+ breast cancer. Ann. Oncol..

[36] Conte P., Frassoldati A., Bisagni G., Brandes A.A., Donadio M., Garrone O., Piacentini F., Cavanna L., Giotta F., Aieta M. (2018). Nine weeks versus 1 year adjuvant trastuzumab in combination with chemotherapy: Final results of the phase III randomized Short-HER studydouble dagger. Ann. Oncol..

[37] Prat A., Guarneri V., Pare L., Griguolo G., Pascual T., Dieci M.V., Chic N., Gonzalez-Farre B., Frassoldati A., Sanfeliu E. (2020). A multivariable prognostic score to guide systemic therapy in early-stage HER2-positive breast cancer: A retrospective study with an external evaluation. Lancet Oncol..

[38] Solinas C., Ceppi M., Lambertini M., Scartozzi M., Garaud S., Fumagalli D., De Azambuja E., Salgado R., Willard-Gallo K., Ignatiadis M. (2017). Tumor infiltrating lymphocytes in HER2 positive breast cancer patients treated with neoadjuvant chemotherapy plus trastuzumab, lapatinib or the combination: A meta-analysis of published randomized clinical trials. Ann. Oncol..

[39] Fumagalli D., Venet D., Ignatiadis M., Azim H.A., Maetens M., Rothe F., Salgado R., Bradbury I., Pusztai L., Harbeck N. (2017). RNA Sequencing to Predict Response to Neoadjuvant Anti-HER2 Therapy: A Secondary Analysis of the NeoALTTO Randomized Clinical Trial. JAMA Oncol..

[40] Prat A., Bianchini G., Thomas M., Belousov A., Cheang M.C., Koehler A., Gomez P., Semiglazov V., Eiermann W., Tjulandin S. (2014). Research-based PAM50 subtype predictor identifies higher responses and improved survival outcomes in HER2-positive breast cancer in the NOAH study. Clin. Cancer Res..

[41] Prat A., Carey L.A., Adamo B., Vidal M., Tabernero J., Cortes J., Parker J.S., Perou C.M., Baselga J. (2014). Molecular features and survival outcomes of the intrinsic subtypes within HER2-positive breast cancer. J. Natl. Cancer Inst..

[42] Dieci M.V., Prat A., Tagliafico E., Pare L., Ficarra G., Bisagni G., Piacentini F., Generali D.G., Conte P., Guarneri V. (2016). Integrated evaluation of PAM50 subtypes and immune modulation of pCR in HER2-positive breast cancer patients treated with chemotherapy and HER2-targeted agents in the CherLOB trial. Ann. Oncol..

[43] Nuciforo P., Pascual T., Cortes J., Llombart-Cussac A., Fasani R., Pare L., Oliveira M., Galvan P., Martinez N., Bermejo B. (2018). A predictive model of pathologic response based on tumor cellularity and tumor-infiltrating lymphocytes (CelTIL) in HER2-positive breast cancer treated with chemo-free dual HER2 blockade. Ann. Oncol..

[44] Guarneri V., Dieci M.V., Bisagni G., Brandes A.A., Frassoldati A., Cavanna L., Musolino A., Giotta F., Rimanti A., Garrone O. (2020). PIK3CA Mutation in the ShortHER Randomized Adjuvant Trial for Patients with Early HER2(+) Breast Cancer: Association with Prognosis and Integration with PAM50 Subtype. Clin. Cancer Res..

[45] Loi S., Sirtaine N., Piette F., Salgado R., Viale G., Van Eenoo F., Rouas G., Francis P., Crown J.P., Hitre E. (2013). Prognostic and predictive value of tumor-infiltrating lymphocytes in a phase III randomized adjuvant breast cancer trial in node-positive breast cancer comparing the addition of docetaxel to doxorubicin with doxorubicin-based chemotherapy: BIG 02-98. J. Clin. Oncol..

[46] Dieci M.V., Mathieu M.C., Guarneri V., Conte P., Delaloge S., Andre F., Goubar A. (2015). Prognostic and predictive value of tumor-infiltrating lymphocytes in two phase III randomized adjuvant breast cancer trials. Ann. Oncol..

[47] Ali H.R., Provenzano E., Dawson S.J., Blows F.M., Liu B., Shah M., Earl H.M., Poole C.J., Hiller L., Dunn J.A. (2014). Association between CD8+ T-cell infiltration and breast cancer survival in 12,439 patients. Ann. Oncol..

[48] Sobral-Leite M., Salomon I., Opdam M., Kruger D.T., Beelen K.J., van der Noort V., van Vlierberghe R.L.P., Blok E.J., Giardiello D., Sanders J. (2019). Cancer-immune interactions in ER-positive breast cancers: PI3K pathway alterations and tumor-infiltrating lymphocytes. Breast Cancer Res..

[49] Criscitiello C., Vingiani A., Maisonneuve P., Viale G., Viale G., Curigliano G. (2020). Tumor-infiltrating lymphocytes (TILs) in ER+/HER2- breast cancer. Breast Cancer Res. Treat.

[50] Mahmoud S.M., Paish E.C., Powe D.G., Macmillan R.D., Grainge M.J., Lee A.H., Ellis I.O., Green A.R. (2011). Tumor-infiltrating CD8+ lymphocytes predict clinical outcome in breast cancer. J. Clin. Oncol..

[51] Brown J.R., Wimberly H., Lannin D.R., Nixon C., Rimm D.L., Bossuyt V. (2014). Multiplexed quantitative analysis of CD3, CD8, and CD20 predicts response to neoadjuvant chemotherapy in breast cancer. Clin. Cancer Res..

[52] Lee H.J., Seo J.Y., Ahn J.H., Ahn S.H., Gong G. (2013). Tumor-associated lymphocytes predict response to neoadjuvant chemotherapy in breast cancer patients. J. Breast Cancer.

[53] Hornychova H., Melichar B., Tomsova M., Mergancova J., Urminska H., Ryska A. (2008). Tumor-infiltrating lymphocytes predict response to neoadjuvant chemotherapy in patients with breast carcinoma. Cancer Investig..

[54] Oda N., Shimazu K., Naoi Y., Morimoto K., Shimomura A., Shimoda M., Kagara N., Maruyama N., Kim S.J., Noguchi S. (2012). Intratumoral regulatory T cells as an independent predictive factor for pathological complete response to neoadjuvant paclitaxel followed by 5-FU/epirubicin/cyclophosphamide in breast cancer patients. Breast Cancer Res. Treat.

[55] Garcia-Martinez E., Gil G.L., Benito A.C., Gonzalez-Billalabeitia E., Conesa M.A., Garcia Garcia T., Garcia-Garre E., Vicente V., Ayala de la Pena F. (2014). Tumor-infiltrating immune cell profiles and their change after neoadjuvant chemotherapy predict response and prognosis of breast cancer. Breast Cancer Res..

[56] Liu S., Lachapelle J., Leung S., Gao D., Foulkes W.D., Nielsen T.O. (2012). CD8+ lymphocyte infiltration is an independent favorable prognostic indicator in basal-like breast cancer. Breast Cancer Res..

[57] Schmidt M., Weyer-Elberich V., Hengstler J.G., Heimes A.S., Almstedt K., Gerhold-Ay A., Lebrecht A., Battista M.J., Hasenburg A., Sahin U. (2018). Prognostic impact of CD4-positive T cell subsets in early breast cancer: A study based on the FinHer trial patient population. Breast Cancer Res..

[58] Gruosso T., Gigoux M., Manem V.S.K., Bertos N., Zuo D., Perlitch I., Saleh S.M.I., Zhao H., Souleimanova M., Johnson R.M. (2019). Spatially distinct tumor immune microenvironments stratify triple-negative breast cancers. J. Clin. Investig..

[59] West N.R., Milne K., Truong P.T., Macpherson N., Nelson B.H., Watson P.H. (2011). Tumor-infiltrating lymphocytes predict response to anthracycline-based chemotherapy in estrogen receptor-negative breast cancer. Breast Cancer Res..

[60] Seo A.N., Lee H.J., Kim E.J., Kim H.J., Jang M.H., Lee H.E., Kim Y.J., Kim J.H., Park S.Y. (2013). Tumour-infiltrating CD8+ lymphocytes as an independent predictive factor for pathological complete response to primary systemic therapy in breast cancer. Br. J. Cancer.

[61] Badr N.M., Berditchevski F., Shaaban A.M. (2020). The Immune Microenvironment in Breast Carcinoma: Predictive and Prognostic Role in the Neoadjuvant Setting. Pathobiology.

[62] Bottai G., Raschioni C., Losurdo A., Di Tommaso L., Tinterri C., Torrisi R., Reis-Filho J.S., Roncalli M., Sotiriou C., Santoro A. (2016). An immune stratification reveals a subset of PD-1/LAG-3 double-positive triple-negative breast cancers. Breast Cancer Res..

[63] Hou Y., Nitta H., Wei L., Banks P.M., Parwani A.V., Li Z. (2018). Evaluation of Immune Reaction and PD-L1 Expression Using Multiplex Immunohistochemistry in HER2-Positive Breast Cancer: The Association With Response to Anti-HER2 Neoadjuvant Therapy. Clin. Breast Cancer.

[64] Asano Y., Kashiwagi S., Goto W., Kurata K., Noda S., Takashima T., Onoda N., Tanaka S., Ohsawa M., Hirakawa K. (2016). Tumour-infiltrating CD8 to FOXP3 lymphocyte ratio in predicting treatment responses to neoadjuvant chemotherapy of aggressive breast cancer. Br. J. Surg..

[65] De Angelis C., Nagi C., Hoyt C.C., Liu L., Roman K., Wang C., Zheng Y., Veeraraghavan J., Sethunath V., Nuciforo P. (2020). Evaluation of the Predictive Role of Tumor Immune Infiltrate in Patients with HER2-Positive Breast Cancer Treated with Neoadjuvant Anti-HER2 Therapy without Chemotherapy. Clin. Cancer Res..

[66] Al-Saleh K., Abd El-Aziz N., Ali A., Abozeed W., Abd El-Warith A., Ibraheem A., Ansari J., Al-Rikabi A., Husain S., Nabholtz J.M. (2017). Predictive and prognostic significance of CD8(+) tumor-infiltrating lymphocytes in patients with luminal B/HER 2 negative breast cancer treated with neoadjuvant chemotherapy. Oncol. Lett..

[67] Goto W., Kashiwagi S., Asano Y., Takada K., Takahashi K., Hatano T., Takashima T., Tomita S., Motomura H., Ohsawa M. (2018). Predictive value of improvement in the immune tumour microenvironment in patients with breast cancer treated with neoadjuvant chemotherapy. ESMO Open.

[68] Bates G.J., Fox S.B., Han C., Leek R.D., Garcia J.F., Harris A.L., Banham A.H. (2006). Quantification of regulatory T cells enables the identification of high-risk breast cancer patients and those at risk of late relapse. J. Clin. Oncol..

[69] Liu S., Foulkes W.D., Leung S., Gao D., Lau S., Kos Z., Nielsen T.O. (2014). Prognostic significance of FOXP3+ tumor-infiltrating lymphocytes in breast cancer depends on estrogen receptor and human epidermal growth factor receptor-2 expression status and concurrent cytotoxic T-cell infiltration. Breast Cancer Res..

[70] Mohammed Z.M., Going J.J., Edwards J., McMillan D.C. (2012). The role of the tumour inflammatory cell infiltrate in predicting recurrence and survival in patients with primary operable breast cancer. Cancer Treat. Rev..

[71] Iglesia M.D., Vincent B.G., Parker J.S., Hoadley K.A., Carey L.A., Perou C.M., Serody J.S. (2014). Prognostic B-cell signatures using mRNA-seq in patients with subtype-specific breast and ovarian cancer. Clin. Cancer Res..

[72] Denkert C., Loibl S., Noske A., Roller M., Muller B.M., Komor M., Budczies J., Darb-Esfahani S., Kronenwett R., Hanusch C. (2010). Tumor-associated lymphocytes as an independent predictor of response to neoadjuvant chemotherapy in breast cancer. J. Clin. Oncol..

[73] Szekely B., Bossuyt V., Li X., Wali V.B., Patwardhan G.A., Frederick C., Silber A., Park T., Harigopal M., Pelekanou V. (2018). Immunological differences between primary and metastatic breast cancer. Ann. Oncol..

[74] Liu S., Chen B., Burugu S., Leung S., Gao D., Virk S., Kos Z., Parulekar W.R., Shepherd L., Gelmon K.A. (2017). Role of Cytotoxic Tumor-Infiltrating Lymphocytes in Predicting Outcomes in Metastatic HER2-Positive Breast Cancer: A Secondary Analysis of a Randomized Clinical Trial. JAMA Oncol..

[75] Schmid P., Adams S., Rugo H.S., Schneeweiss A., Barrios C.H., Iwata H., Dieras V., Hegg R., Im S.A., Shaw Wright G. (2018). Atezolizumab and Nab-Paclitaxel in Advanced Triple-Negative Breast Cancer. N. Engl. J. Med..

[76] Cortes J., Cescon D., Rugo H., Nowecki Z., Im S., Yusof M., Gallardo C., Lipatov O., Barrios C., Holgado E. (2020). KEYNOTE-355: Randomized, double-blind, phase III study of pembrolizumab + chemotherapy versus placebo + chemotherapy for previously untreated locally recurrent inoperable or metastatic triple-negative breast cancer. J. Clin. Oncol..

[77] Miles D., Gligorov J., André F., Cameron D., Schneeweiss A., Barrios C., Xu B., Wardley A., Kaen D., Andrade L. (2020). Primary results from IMpassion131, a double-blind placebo-controlled randomised phase III trial of first-line paclitaxel (PAC) ± atezolizumab (atezo) for unresectable locally advanced/metastatic triple-negative breast cancer (mTNBC). Ann. Oncol..

[78] Miglietta F., Griguolo G., Guarneri V., Dieci M.V. (2019). Programmed Cell Death Ligand 1 in Breast Cancer: Technical Aspects, Prognostic Implications, and Predictive Value. Oncologist.

[79] Schmid P., Cortes J., Pusztai L., McArthur H., Kummel S., Bergh J., Denkert C., Park Y.H., Hui R., Harbeck N. (2020). Pembrolizumab for Early Triple-Negative Breast Cancer. N. Engl. J. Med..

[80] Loibl S., Untch M., Burchardi N., Huober J., Sinn B.V., Blohmer J.U., Grischke E.M., Furlanetto J., Tesch H., Hanusch C. (2019). A randomised phase II study investigating durvalumab in addition to an anthracycline taxane-based neoadjuvant therapy in early triple-negative breast cancer: Clinical results and biomarker analysis of GeparNuevo study. Ann. Oncol..

[81] Mittendorf E., Barrios H., Harbeck N., Jung K., Miles D., Saji S., Zhang H., Duc A., Rafii S., Lai C. (2017). IMpassion031: A phase III study comparing neoadjuvant atezolizumab (atezo) vs placebo in combination with anthracycline/nab-paclitaxel (nab-pac)–based chemotherapy in early triple-negative breast cancer (eTNBC). Ann. Oncol..

[82] Mittendorf E.A., Zhang H., Barrios C.H., Saji S., Jung K.H., Hegg R., Koehler A., Sohn J., Iwata H., Telli M.L. (2020). Neoadjuvant atezolizumab in combination with sequential nab-paclitaxel and anthracycline-based chemotherapy versus placebo and chemotherapy in patients with early-stage triple-negative breast cancer (IMpassion031): A randomised, double-blind, phase 3 trial. Lancet.

[83] Schmid P., Salgado R., Park Y.H., Munoz-Couselo E., Kim S.B., Sohn J., Im S.A., Foukakis T., Kuemmel S., Dent R. (2020). Pembrolizumab plus chemotherapy as neoadjuvant treatment of high-risk, early-stage triple-negative breast cancer: Results from the phase 1b open-label, multicohort KEYNOTE-173 study. Ann. Oncol..

[84] Gianni L., Huang C.S., Egle D., Bermejo B., Zamagni C., Thill M., Anton A., Zambelli S., Bianchini G., Russo S. (2020). Pathologic Complete Response to Neoadjuvant Treatment with or without Atezolizumab in Triple-Negative, Early high-Risk and Locally Advanced Breast Cancer. NeoTRIPaPDL1 Michelangelo Randomized Study.

[85] Bianchini G., Huang C., Egle D., Bermejo B., Zamagni C., Thill M., Anton A., Zambelli S., Russo S., Ciruelos E. (2020). Tumour infiltrating lymphocytes (TILs), PD-L1 expression and their dynamics in the NeoTRIPaPDL1 trial. Ann. Oncol..

[86] Emens L., Loi S., Rugo H., Schneeweiss A., Dieras V., Iwata H., Barrios C., Nechaeva M., Molinero L., Nguyen A. IMpassion130: Efficacy in immune biomarker subgroup from the global, randomized, double-blind, placebo-controlled, phase III study of atezolizumab + nab-paclitaxel in patients with treatment-naive, locally advanced or metastatic triple-negative breast cancer. Presented at the 2018 San Antonio Breast Cancer Sympsium.

[87] Loi S., Adams S., Schmid P., Cortes J., Cescon D., Winer E., Toppmeyer D., Rugo H., De Laurentiis M., Nanda R. Relationship between tumor infiltrating lymphocyte levels and response to pembrolizumab in metastatic triple-negative breast cancer: Results from Keynote-086 trial. Presented at the European Society of Medical Oncology (ESMO) 2017 Congress.

[88] Cortes J., Lipatov O., Im S., Goncalves A., Lee K., Schmid P., Tamura K., Testa L., Witzel I., Ohtani S. KEYNOTE-119: Phase III study of pembrolizumab (pembro) versus single-agent chemotherapy (chemo) for metastatic triple negative breast cancer (mTNBC). Proceedings of the ESMO Congress 2019.

[89] Loi L., Winer E., Lipatov O., Im S., Goncalves A., Cortes J., Lee K., Schmid P., Testa L., Witzel I. (2020). Relationship between Tumor-Infiltrating Lymphocytes (TILs) and Outcomes in the KEYNOTE-119 Study of Pembrolizumab vs Chemotherapy for Previously Treated Metastatic Triple-Negative Breast Cancer (mTNBC).

[90] Emens L.A., Esteva F.J., Beresford M., Saura C., De Laurentiis M., Kim S.B., Im S.A., Wang Y., Salgado R., Mani A. (2020). Trastuzumab emtansine plus atezolizumab versus trastuzumab emtansine plus placebo in previously treated, HER2-positive advanced breast cancer (KATE2): A phase 2, multicentre, randomised, double-blind trial. Lancet Oncol.

[91] Emens L., Esteva F., Beresford M., Saura C., De Laurentiis M., Kim S., Im S., Wang Y., Mani A., Shah J. (2019). Overall Survival (OS) in KATE2, a phase 2 study of programmed death ligand 1 (PD-L1) inhibitor Atezolizumab (Atezo) + Trastuzumab Emtansine (T-DM1) vs placebo (PBO) in previously treated HER2+ advanced breast cancer. Ann. Oncol..

[92] Dieci M., Guarneri V., Bisagni G., Tosi A., Musolino A., Spazzapan S., Moretti G., Vernaci G., Giarratano T., Lo Mele M. (2020). Neoadjuvant chemotherapy and immunotherapy in Luminal B BC: Results of the phase II GIADA trial. Ann. Oncol..

[93] Gabrilovich D.I., Ishida T., Nadaf S., Ohm J.E., Carbone D.P. (1999). Antibodies to vascular endothelial growth factor enhance the efficacy of cancer immunotherapy by improving endogenous dendritic cell function. Clin. Cancer Res..

[94] Bell D., Chomarat P., Broyles D., Netto G., Harb G.M., Lebecque S., Valladeau J., Davoust J., Palucka K.A., Banchereau J. (1999). In breast carcinoma tissue, immature dendritic cells reside within the tumor, whereas mature dendritic cells are located in peritumoral areas. J. Exp. Med..

[95] Lee H., Lee H.J., Song I.H., Bang W.S., Heo S.H., Gong G., Park I.A. (2018). CD11c-Positive Dendritic Cells in Triple-negative Breast Cancer. In Vivo.

[96] Lespagnard L., Gancberg D., Rouas G., Leclercq G., de Saint-Aubain Somerhausen N., Di Leo A., Piccart M., Verhest A., Larsimont D. (1999). Tumor-infiltrating dendritic cells in adenocarcinomas of the breast: A study of 143 neoplasms with a correlation to usual prognostic factors and to clinical outcome. Int. J. Cancer.

[97] Treilleux I., Blay J.Y., Bendriss-Vermare N., Ray-Coquard I., Bachelot T., Guastalla J.P., Bremond A., Goddard S., Pin J.J., Barthelemy-Dubois C. (2004). Dendritic cell infiltration and prognosis of early stage breast cancer. Clin. Cancer Res..

[98] Iwamoto M., Shinohara H., Miyamoto A., Okuzawa M., Mabuchi H., Nohara T., Gon G., Toyoda M., Tanigawa N. (2003). Prognostic value of tumor-infiltrating dendritic cells expressing CD83 in human breast carcinomas. Int. J. Cancer.

[99] Lu J., Ma L. (2020). The role of tumor-associated macrophages in the development, metastasis and treatment of breast cancer. Pathol. Res. Pract..

[100] Qiu S.Q., Waaijer S.J.H., Zwager M.C., de Vries E.G.E., van der Vegt B., Schroder C.P. (2018). Tumor-associated macrophages in breast cancer: Innocent bystander or important player?. Cancer Treat. Rev..

[101] Tiainen S., Tumelius R., Rilla K., Hamalainen K., Tammi M., Tammi R., Kosma V.M., Oikari S., Auvinen P. (2015). High numbers of macrophages, especially M2-like (CD163-positive), correlate with hyaluronan accumulation and poor outcome in breast cancer. Histopathology.

[102] Brufsky A.M., Davidson N.E. (2016). Multiparametric Genomic Assays for Breast Cancer: Time for the Next Generation?. Clin. Cancer Res..

[103] Mahmoud S.M., Lee A.H., Paish E.C., Macmillan R.D., Ellis I.O., Green A.R. (2012). Tumour-infiltrating macrophages and clinical outcome in breast cancer. J. Clin Pathol..

[104] Yuan Z.Y., Luo R.Z., Peng R.J., Wang S.S., Xue C. (2014). High infiltration of tumor-associated macrophages in triple-negative breast cancer is associated with a higher risk of distant metastasis. Oncol. Targets Ther..

[105] Gwak J.M., Jang M.H., Kim D.I., Seo A.N., Park S.Y. (2015). Prognostic value of tumor-associated macrophages according to histologic locations and hormone receptor status in breast cancer. PLoS ONE.

[106] Bense R.D., Sotiriou C., Piccart-Gebhart M.J., Haanen J.B.A.G., van Vugt M.A.T.M., de Vries E.G.E., Schroder C.P., Fehrmann R.S.N. (2016). Relevance of Tumor-Infiltrating Immune Cell Composition and Functionality for Disease Outcome in Breast Cancer. J. Natl. Cancer Inst..

[107] Ali H.R., Chlon L., Pharoah P.D., Markowetz F., Caldas C. (2016). Patterns of Immune Infiltration in Breast Cancer and Their Clinical Implications: A Gene-Expression-Based Retrospective Study. PLoS Med..

[108] Li D., Ji H., Niu X., Yin L., Wang Y., Gu Y., Wang J., Zhou X., Zhang H., Zhang Q. (2020). Tumor-associated macrophages secrete CC-chemokine ligand 2 and induce tamoxifen resistance by activating PI3K/Akt/mTOR in breast cancer. Cancer Sci..

[109] Sousa S., Brion R., Lintunen M., Kronqvist P., Sandholm J., Monkkonen J., Kellokumpu-Lehtinen P.L., Lauttia S., Tynninen O., Joensuu H. (2015). Human breast cancer cells educate macrophages toward the M2 activation status. Breast Cancer Res..

[110] Zhang Y., Cheng S., Zhang M., Zhen L., Pang D., Zhang Q., Li Z. (2013). High-infiltration of tumor-associated macrophages predicts unfavorable clinical outcome for node-negative breast cancer. PLoS ONE.

[111] Kaewkangsadan V., Verma C., Eremin J.M., Cowley G., Ilyas M., Satthaporn S., Eremin O. (2017). The Differential Contribution of the Innate Immune System to a Good Pathological Response in the Breast and Axillary Lymph Nodes Induced by Neoadjuvant Chemotherapy in Women with Large and Locally Advanced Breast Cancers. J. Immunol. Res..

[112] Pelekanou V., Villarroel-Espindola F., Schalper K.A., Pusztai L., Rimm D.L. (2018). CD68, CD163, and matrix metalloproteinase 9 (MMP-9) co-localization in breast tumor microenvironment predicts survival differently in ER-positive and -negative cancers. Breast Cancer Res..

[113] Matikas A., Lovrot J., Ramberg A., Eriksson M., Lindsten T., Lekberg T., Hedenfalk I., Loman N., Bergh J., Hatschek T. (2018). Dynamic evaluation of the immune infiltrate and immune function genes as predictive markers for neoadjuvant chemotherapy in hormone receptor positive, HER2 negative breast cancer. Oncoimmunology.

[114] Ascierto M.L., Idowu M.O., Zhao Y., Khalak H., Payne K.K., Wang X.Y., Dumur C.I., Bedognetti D., Tomei S., Ascierto P.A. (2013). Molecular signatures mostly associated with NK cells are predictive of relapse free survival in breast cancer patients. J. Transl. Med..

[115] Verma C., Kaewkangsadan V., Eremin J.M., Cowley G.P., Ilyas M., El-Sheemy M.A., Eremin O. (2015). Natural killer (NK) cell profiles in blood and tumour in women with large and locally advanced breast cancer (LLABC) and their contribution to a pathological complete response (PCR) in the tumour following neoadjuvant chemotherapy (NAC): Differential restoration of blood profiles by NAC and surgery. J. Transl. Med..

[116] Muntasell A., Cabo M., Servitja S., Tusquets I., Martinez-Garcia M., Rovira A., Rojo F., Albanell J., Lopez-Botet M. (2017). Interplay between Natural Killer Cells and Anti-HER2 Antibodies: Perspectives for Breast Cancer Immunotherapy. Front. Immunol..

[117] Solinas C., Boisson A., Brown D., de Wind R., van den Eynden G., Garaud S., Buisseret L., Naveaux C., Sotiriou C., Larsimont D. (2016). ESMO 2016 Tumor infiltrating lymphocytes and tertiary lymphoid structures in paired primary tumors and metastases from breast cancer patients. Ann. Oncol..

[118] Klauschen F., Muller K.R., Binder A., Bockmayr M., Hagele M., Seegerer P., Wienert S., Pruneri G., de Maria S., Badve S. (2018). Scoring of tumor-infiltrating lymphocytes: From visual estimation to machine learning. Semin. Cancer Biol..

[119] Rugo H., Loi S., Adams S., Schmid P., Schneeweiss A., Barrios C., Iwata H., Diéras V., Winer E., Peeters D. (2019). Performance of PD-L1 immunohistochemistry assays in unresectable locally advanced or metastatic triple-negative breast cancer: Post hoc analysis of IMpassion130. Ann. Oncol..

